# Navigating the Bayes maze: The psychologist's guide to Bayesian statistics, a hands‐on tutorial with R code

**DOI:** 10.1002/ijop.13271

**Published:** 2024-12-19

**Authors:** Udi Alter, Miranda A. Too, Robert A. Cribbie

**Affiliations:** ^1^ Department of Psychology York University Toronto ON Canada

**Keywords:** Bayesian statistics, Bayesian inference, tutorial, R, brms

## Abstract

Bayesian statistics has gained substantial popularity in the social sciences, particularly in psychology. Despite its growing prominence in the psychological literature, many researchers remain unacquainted with Bayesian methods and their advantages. This tutorial addresses the needs of curious applied psychology researchers and introduces Bayesian analysis as an accessible and powerful tool. We begin by comparing Bayesian and frequentist approaches, redefining fundamental terms from both perspectives with practical illustrations. Our exploration of Bayesian statistics includes Bayes's Theorem, likelihood, prior and posterior distributions, various prior types, and Markov‐Chain Monte Carlo (MCMC) methods, supplemented by graphical aids for clarity. To bridge theory and practice, we employ a psychological research example with real, open data. We analyse the data using both frequentist and Bayesian approaches, providing R code and comprehensive supporting information, and emphasising best practices for interpretation and reporting. We discuss and demonstrate how to interpret parameter estimates and credible intervals, among other essential topics. Throughout, we maintain an accessible and user‐friendly language, focusing on practical implications, intuitive examples, and actionable recommendations.

Bayesian statistics, although not a novel school of thought, has recently surged in popularity within the social sciences. Particularly in psychology, Bayesian methods and inference have been gaining a fair amount of traction, but many applied researchers remain unfamiliar or inexperienced with these analyses or the advantages they offer. Being Bayesian literate opens the door to powerful tools for probabilistic reasoning and a greater capacity for flexible decision‐making in the face of uncertainty, all of which are applicable across diverse research contexts and statistical techniques. Fortunately, the contemporary landscape of computational power and accessible software has made Bayesian analysis much more adaptable, convenient, and practical than ever before. What is not always so adaptable, convenient, and practical are textbooks and journal articles, which often assume prior statistical knowledge or include a high Greek letters‐to‐text ratio. In this tutorial, we answer the call of many curious psychology researchers and students. This paper is designed with the applied researcher in mind and assumes minimal statistical proficiency; it is a *gentle* introduction to Bayesian inference with an emphasis on the fundamental concepts and applications using clear examples and annotated R code.

## CLEARING THE BAYES HAZE

We cannot talk about (Bayesian) statistics without first talking about probability. So far you have likely been using a frequentist approach to inferential statistics—otherwise, you wouldn't be reading this tutorial. Bayesian statistics has a few fundamental differences in perspective and thinking. We start with one of the most critical differences, the definition of probability.

### Defining probability

#### Frequentist probability

From the frequentist perspective, “probability” is simply the *frequency* at which an event has occurred (hence the name). If we repeatedly conduct an experiment (e.g., toss a coin) *N* times, we can count how many times the experiment resulted in a specific outcome (e.g., coin lands on “heads”) and divide it by *N*. The probability, denoted Pr, of an event is then the *proportion* of times the event (*E*) has occurred, as *N* approaches infinity. Or, in equation form,

(1) $$\mathrm{Pr}\left(E\right)=\lim_{N\to \infty }\frac{\text{number of times event}\;E\;\text{occurred}}{N}$$

For example, we can estimate the probability of a new therapy being effective by administering the therapy to *N* random individuals. The *estimated* probability that this new therapy is effective is simply the number of times the therapy was effective (by some definition of “effective”) divided by the total number of times the therapy was administered. Here, we emphasise the estimation of the probability because, realistically, we cannot conduct this experiment ad infinitum. Instead, we make an inference about the true (yet, unknown) probability of the treatment being effective from the sample we gathered.

Hypothetically, if we did administer this new therapy indefinitely and calculate the proportion of times the treatment was effective, we would get the true probability. The true probability in this example refers to the population parameter—the thing we want to know—which, from the frequentist's lens, is a *single*, *objective* and *fixed* value, but unknown to us. In running an experiment, we calculate the parameter of interest from a sample and use this value as a proxy (i.e., estimate) for the unknown population parameter. The population parameter is a *single* value because there is only one possible value that the true parameter can take. The population parameter is also *objective* because it is unequivocally true even though we usually cannot know its value. Finally, the population parameter is also *fixed*; it is a constant value that does not vary at the time of observation. As you will see next, Bayesian philosophy has quite a different take on probability and the parameter of interest.

#### Bayesian probability and inference

From a Bayesian perspective, “probability” is defined as a measure of belief, or credibility (Kruschke, 2015), about an event, hypothesis, or parameter. Bayesian probability represents the *strength of our belief* in the truth of a hypothesis, the value of a parameter, or the occurrence of an event, based on available evidence and prior knowledge. Bayesian probability is the marriage of prior beliefs (which could be based on previous knowledge) with new evidence (e.g., the data we collected for an experiment). Returning to our previous example, the probability of a new therapy treatment being effective, from a Bayesian lens, is our degree of belief that the treatment is indeed effective given both the data and prior knowledge.

Our prior knowledge or belief about an event, hypothesis, or parameter is called a *prior probability distribution*, or simply, *prior*, and is denoted Pr(parameter). The prior tells us what is the most credible parameter value without looking at the data. We can then reassess our prior beliefs in light of the new evidence (i.e., data). In essence, this reassessment is simply just reallocating the crediblities of each parameter value across the entire range of possible values (Kruschke, 2015). The reallocation of credibilities across possibilities is what makes a Bayesian inference, it is our *updated belief* about the parameter in question. Our updated or revised belief, which incorporates both the prior and the new evidence, is called the *posterior probability distribution*, or simply, the *posterior*, and is denoted Pr(parameter|data) which reads: the probability of the parameter, given the data. The posterior distribution *is* our Bayesian inference which is built from the model, the data, and the prior Gelman, Hill and Vehtari  (2020).

Because Bayesian probability reflects *our* degree of belief, it is therefore *subjective*, unlike a frequentist probability. Another noteworthy philosophical (and mathematical) difference is that, in frequentist statistics, the population parameter we estimate is believed to be a single, fixed value, whereas in Bayesian statistics the parameter can take *many* values (also called a “random variable”) and can *change* or *update* as new evidence becomes available. This shift in perspective involves transitioning from viewing the parameter of interest as a static value to treating it as a distribution. If we were to plot this distribution (and we do in Figure 2), the *x*‐axis would represent the full range of possible parameter values, whereas the *y*‐axis would indicate the probability (or probability density) assigned each of these values.

You then might wonder: if the parameter of interest can take more than a single value, how do we know which value to use and interpret? The answer to this question is illustrated in the research example below. But, for now, know that the “workable” value we usually use is the one we believe to be the most probable; that is, the value that has the highest credibility given both prior information and the data. To find the most credible value, we can solve for the possible parameter value for which the distribution density is highest or calculate the mean or median of the posterior distribution (more on this soon).

### Bayes's Theorem

**Figure 1:**
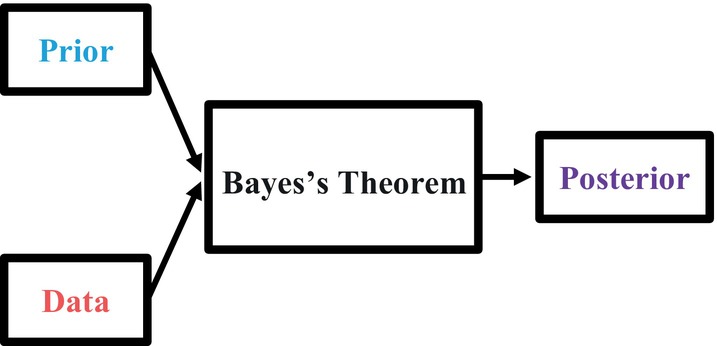
Roadmap to Bayesian inference. *Note*: The prior represents known (or believed to be known) information about the parameter of interest and, together with the data, form the posterior distribution, the updated belief that incorporates both the prior and the new evidence.

Earlier, we mentioned two important principles: (1) Bayesian probability is the marriage of prior beliefs with new evidence (i.e., data), and (2) the parameter value can change or update as new evidence becomes available. These two principles are closely related, and they refer to the process by which we “glue” the prior with the data and update our beliefs about the parameter of interest. The roadmap for this process is illustrated in Figure 1.

Figure 1 shows the different components of Bayesian inference and how they fit together to form the final product, the posterior distribution. In Figure 1, how the prior and data combine is represented by both the literal and figurative black box labelled Bayes's Theorem. Inside this box is the following equation, which is the very root of Bayesian statistics:

(2) $$\mathrm{Pr}\left(\text{parameter}|\text{data}\right)=\frac{\mathrm{Pr}\left(\text{parameter}\right)\cdot \mathrm{Pr}\left(\text{data}|\text{parameter}\right)}{\mathrm{Pr}\left(\text{data}\right)}.$$

The interpretation of Bayes's Theorem, as given by the equation above, is as follows: Consider that the parameter above represents some uncertain proposition (a hypothesis, if you will; e.g., in a linear regression model, a population regression coefficient equals zero, *b* = 0) and we wish to establish the strength of our belief in this hypothesis in light of the data and what we already know about this relationship. In preparation for estimating our model, we specify our strength of belief in—the Bayesian probability of—the hypothesis, without looking at the data; this is the *prior*, Pr(parameter). For example, Pr(*b* = 0). We then multiply it by the data. More, precisely, we multiply the prior by some function we created from the data called the *likelihood* which represents how likely it is to observe the data if the hypothesis is indeed true. The likelihood is denoted by Pr(data|parameter). For example, Pr(data|*b* = 0).

**Figure 2:**
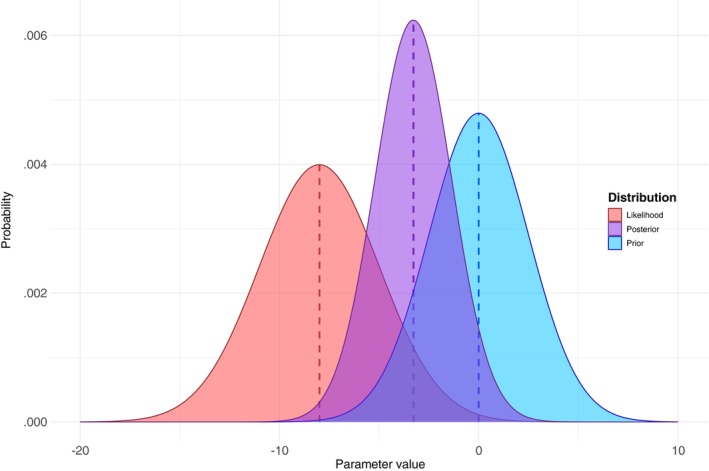
Visualisation of how the prior and likelihood distributions combine to form the posterior probability distribution. *Note*: The prior probability distribution, Pr(parameter), in blue, and the likelihood of the data (the probability of the data given the parameter), Pr(data|parameter), in red, are combined to get the posterior probability distribution, Pr(parameter|data), in purple. The mean of the posterior (vertical purple line) is between the means of the prior and the likelihood (vertical blue and red lines, respectively) indicating both their contributions to the posterior.

The product of the prior and likelihood is divided by the probability of observing the data, under any circumstances, Pr(data). The result is the *posterior* probability distribution, our updated degree of belief about the parameter of interest and therefore about the truth of the hypothesis in light of new data, Pr(parameter|data). For example, Pr(*b* = 0|data). The combination of the prior with the likelihood of the data is illustrated in Figure 2: the posterior distribution (in purple) is located between the likelihood of the data (in red) and the prior distribution (in blue), indicating that it is the consequence of combining the two components—the child from the marriage between the prior and the data. Next, we discuss the different types of priors.

### A prior to priors

#### Noninformative (flat) priors

As the name suggests, *noninformative priors* are (typically) not informative. There are multiple types of noninformative prior distributions, but their distinction is beyond the scope of this tutorial. Noninformative priors are sometimes called *flat priors* because their probability density function looks like a horizontal line when plotted on a graph. This type of distribution is classified as a uniform distribution because every potential value the parameter can take has an equal (hence, uniform) probability. For this reason, noninformative priors reflect ignorance about the parameters to which we assign this prior (Fox, 2022). Generally, it is not recommended to use noninformative priors unless we truly know nothing and have no prior beliefs about the likely values of the parameter (Johnson et al., 2022; McElreath, 2020). In fact, using a noninformative prior will, in some cases, result in a posterior that will be identical to the likelihood distribution (which will yield parameter estimates that are not different from a frequentist inference using maximum‐likelihood estimation). To that extent, using Bayesian inference might be unwarranted because, at the heart of Bayesian statistics, is the idea of priors.

#### Weakly informative (default) priors

Oftentimes, we do not have a strong conviction about our parameter of interest but, instead, have just a broad idea or perhaps some known constraints (e.g., the association between hours spent studying for a math test and its grade will be positive). In such situations, we can use *weakly informative*, also called *vaguely informative* or *default priors*. Weakly informative priors convey some general information or constraints about the parameter of interest without strongly influencing the posterior results; these priors strike a balance between incorporating some previous beliefs while weighing down any strong biases. This is particularly useful when there is limited prior information or when the goal is to let the data “dominate” the results (Fox, 2022) Gelman, Hill, and Vehtari (2020).

#### Informative priors

In contrast to weakly informative or noninformative priors, informative priors convey more deliberate and specific information about the parameter of interest. Informative priors are usually determined based on established knowledge, results from previous studies, or experts' opinions and, unlike default or flat priors, will typically have a stronger influence on the resulting posterior distribution (Fox, 2022). Informative priors are especially helpful with smaller sample sizes because the intended credible parameter space is reduced, allowing for a narrower interval of uncertainty surrounding our inference (Kruschke, 2015).

### Normalisation constant and conjugate priors

You might have noticed that Figure 2 does not include the denominator in Equation 2. The denominator in Equation 2, Pr(data), is the unconditional probability of observing the data. This term is a normalisation constant which is calculated by considering all possible ways that the data could occur, regardless of the specific hypothesis or parameter value. The normalisation constant in Bayes's Theorem plays a role in making the probabilities (as displayed on the *y*‐axis in Figure 2) consistent and meaningful by ensuring they sum up to 1; the normalisation constant converts our resulting distribution to a *probability* distribution with which we are familiar and can easily work.

Truth be told, it can get quite difficult to evaluate Pr(data) because, with continuous variables, we would need to sum the probabilities across an infinite amount of parameter values—we would have to resort to integral calculus, which may not always have closed‐form solutions. This gets exponentially more complicated in multivariate (i.e., multiple parameter) Bayesian estimation situations because every parameter has its own prior (or a joint prior with other parameters), which means we might need to deal with high‐dimensional integrals. Fortunately, it is not always necessary. Actually, the driving relationship in Bayes's Theorem is:

(3) Pr(parameter|data)∝Pr(parameter)·Pr(data|parameter),

which reads: the posterior is proportional to the prior times the likelihood of the data. In some cases, combining the prior distribution with the likelihood distribution results in a posterior which is of the same distributional family as the prior. For example, in Figure 2, the prior is a normal, Gaussian distribution, and together with the likelihood distribution, they form a posterior distribution of the same family (i.e., the posterior is also normally distributed). In cases where the posterior is of the same distributional family as the prior, we say that the prior distribution is a *conjugate* with respect to the likelihood function (Kruschke, 2015).

As it turns out, the mathematics of Bayesian inference is not overly complicated with conjugate priors (Fox, 2022), which saves us (more precisely, the computer) the trouble of calculating the integral of the denominator in Bayes's Theorem. In other cases, however, evaluating the normalisation constant is inevitable, and when there is no closed‐form solution, we can side‐step this difficult task by *approximating* the posterior distribution using a simulated random sampling method called Markov‐Chain Monte Carlo (MCMC), which we briefly turn to next.

### MCMC in two‐and‐a‐half paragraphs

MCMC is an extensive and intricate topic. Many textbooks, dissertations, and journal articles were dedicated to only particular aspects or types of MCMC. We will not be able to do this topic justice without losing sight of this tutorial's goal. We, therefore, treat MCMC in this tutorial a little like a black box but still provide a taste of the ideas behind MCMC in accessible language. Readers are encouraged to peruse Fox (2022), Gelman, Hill, and Vehtari (2020), and Kruschke (2015) for in‐depth review.

MCMC is an umbrella term referring to a set of methods that seek to *approximate* the posterior distribution instead of dealing with the complicated (and sometimes unsolvable) math of calculating the normalisation constant. Approximating the posterior distribution is done by drawing samples (also called iterations) from the posterior function to obtain estimates of its shape, centre, variability and so on. Each sample drawn is viewed as a likely value that the parameter of interest can take. Samples are drawn such that the (*j* + 1)th sample is dependent on (or, “chained” to) the *j*th sample (like links in a chain; hence the name). With enough samples, the posterior distribution can be accurately approximated. Each set of samples, or iterations, represents a single chain. In practice, posterior approximation using MCMC usually consists of multiple chains which are then compared to enhance the reliability and accuracy of the solution. While MCMC is not unique to Bayesian statistics—it “merely provides a high‐resolution pixelated representation of the posterior distribution”—most modern Bayesian analyses use MCMC (Kruschke, 2021, p. 1286).

#### Warm‐up and thinning

Next, we introduce two common practices involved in MCMC estimation designed to improve the accuracy and reliability of the inference. The first is “warm‐up.” *Warm‐up* (also called burn‐in) is an initial phase in the resampling process where samples are discarded to allow the algorithm to stabilise and reach the target distribution; we essentially throw away the first batch of samples (the size of the batch can vary) which we believe might cover an unlikely parameter space. In simple terms, it usually takes the MCMC algorithm time to hone in on a good solution, so we give it a chance to practice (warm‐up) first and then cut that part out (see MCMC diagnostics in the Supporting Information document). Another common practice in MCMC estimation is thinning. *Thinning* aims to reduce autocorrelation, which measures how similar current samples in the chain are to previous ones. High autocorrelation can be problematic because it can prevent samples from efficiently exploring different regions of the parameter space, causing the chain to get “stuck” on specific areas. In thinning, we select every *n*th (e.g., 5th) sample in each chain and discard the rest. Consequently, the remaining samples are more independent from one another which boosts the efficiency and accuracy of the inference.

At this point, we covered the most fundamental concepts we believe are necessary to carry out analyses using Bayesian inference. We now switch gears from theoretical to applied. And, what better way to do this than to begin with a research example.

## RESEARCH EXAMPLE

In the following research example, we use the POLITICS dataset, which includes data from a national survey of U.S. residents. This dataset can be downloaded from the website accompanying Darlington and Hayes's (2016) textbook at www.afhayes.com/regression‐analysis‐and‐linear‐models.html. We encourage readers to download the dataset and follow along with the code snippets weaved into the following sections. For a more comprehensive experience, we strongly recommend consulting the Supporting Information document, available directly at the udialter.github.io/NBM_SupMat.html or on our Open Science Framework (OSF) page at osf.io/2udyq/, where additional open materials are provided. This document offers additional code, detailed explanations, discussions on further topics, and a curated list of valuable resources.

Suppose a team of researchers is interested in understanding how age and education affect political knowledge. To answer this question, the research team surveys *N* = 340 adult respondents between the ages of 18 and 90 years residing in the United States. Participants were asked a series of survey questions about their knowledge of politics and the political process. Responses from the survey questions were aggregated into a composite score representing political knowledge.

To help shed some light on how age and education explain political knowledge, we can use multiple linear regression with political knowledge as the outcome variable, whereas age and education are the regressors. Whether we use frequentist or Bayesian analyses, the linear model is the same:

(4) $$y_{i}=b_{0}+b_{1}\cdot {X_{1}}_{i}+b_{2}\cdot {X_{2}}_{i}+\varepsilon_{i},$$

where *y*_*i*_ is the *i*th (*i* = 1, …, *N*) observed outcome variable value, $b_{0}$ is the population parameter representing the intercept of the regression plane, $b_{1}$ and $b_{2}$ are the population parameters representing the partial slopes (regression coefficients) of the first and second predictor variables, *X*_1*i*_ and *X*_2*i*_ are the *i*th observed values for the first and second predictor variables, and ε_i_ is the error term, $\varepsilon_{i}=y_{i}-{\hat{y}}_{i}$, with ${\hat{y}}_{i}$ being the predicted value on the outcome variable for case *i*. For our research example, we can rewrite the equation above as:

(5) $$\text{pkno}w_{i}=b_{0}+b_{1}\cdot \mathrm{ag}e_{i}+b_{2}\cdot \mathrm{edu}c_{i}+\varepsilon_{i},$$

where *pknow*_*i*_, *age*_*i*_ and *educ*_*i*_, represent political knowledge, age, and education scores for participant *i*, respectively. Descriptive statistics and data visualisations are presented in the Supporting Information document.

## FREQUENTIST APPROACH: MULTIPLE LINEAR REGRESSION USING ORDINARY LEAST SQUARES

To start with something familiar, we first estimate this model using ordinary least squares (OLS) from a frequentist perspective using the lm function from the **stats** package (R Core Team, 2023):

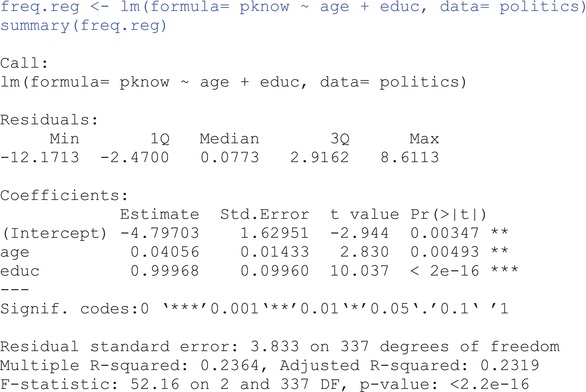

### Interpreting multiple linear regression results using frequentist statistics

We start by assessing how well the model fits the data. Or, better yet, how well age and education simultaneously account for the variability in political knowledge: at the bottom of the output, we see that multiple *R* ^2^ = .2364, suggesting that 23.64% of the variance in political knowledge is explained by the linear combination of the regressors, age and education. With a nominal Type I error rate of α=.05 for this and all subsequent hypothesis tests, an *R* ^2^ = .2364 is statistically different from 0, *F*(2, 337) = 52.16, *p* < .001. We turn next to our estimated parameters of interest, the regression coefficients for age and education, under the heading Coefficients in the output. The intercept is estimated as ${\hat{b}}_{0}=-4.80$, which is the predicted political knowledge score of a hypothetical individual who is 0 years old and has 0 years of education. Of course, this is not a useful estimate because the lowest political knowledge score is 0 (so −4.8 is unobtainable), but also because the researchers are probably not interested in the political knowledge of newborns. If we wanted a realistic interpretation of the intercept, we could have centred age and/or education at specific values (e.g., average age and/or education).

The estimated regression coefficient associated with age is ${\hat{b}}_{\mathrm{age}}=0.04$ and tells us that, if two random individuals with the same level of education (i.e., holding education constant) have a one‐year age difference, we would expect the older individual to have a 0.04‐points higher score in political knowledge. The estimated regression coefficient for education is ${\hat{b}}_{\text{educ}}=0.99$, and can be interpreted similarly: if two random individuals of the same age have a one‐year difference in education, we would expect the more educated individual to have a 0.99‐points higher score on political knowledge.

The Std.Error column specifies the uncertainty (i.e., the standard error) associated with the estimated effects. We can also see that all parameter estimates are statistically significant as noted by the low *p* values under the Pr(>|t|) column. An accurate interpretation of the *p* value of age, for example, is as follows: assuming the partial association between age and political knowledge is exactly 0 (i.e., the null hypothesis is perfectly true), the probability of observing this, or a more extreme effect, is .00493 (i.e., less than 0.5%). Because this probability is so small (i.e., smaller than the nominal Type I error rate), we reject the null hypothesis that *b*_age_ = 0.

Finally, we should also extract and interpret the 95% confidence intervals (CI) for the estimated effects using the following command:

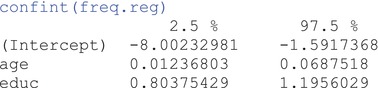

We can interpret the CI as: if we were to sample repeatedly from the population and calculate a CI for each sample, 95% of the intervals are expected to contain the true (population) regression coefficient. The 95% CI for the effect of age, for example, is [0.01, 0.07]—which is a relatively narrow band indicating high precision—and may or may not include the true effect. Keep this interpretation in mind as we transition into Bayesian estimation because the uncertainty about our estimates, as represented by credible intervals (the Bayesian analogue of the frequentist CI), will be interpreted slightly differently. Now, let's see how the results of the same model look when using Bayesian analysis and inference.^1^

## BAYESIAN APPROACH: MULTIPLE LINEAR REGRESSION WITH PRIORS AND MCMC

One of the first steps in Bayesian analysis is choosing a prior for each model parameter. Priors are the hallmark of Bayesian statistics, but they are both a curse and a blessing. On the one hand, they can help us make better inferences if properly selected; on the other hand, they might as easily steer us away from the truth if we make poor prior decisions. Either way, priors (and prior choices) have the potential to profoundly influence the results. Therefore, prior selection should be done deliberately and meticulously, with clear and explicit justification (Kruschke, 2015, 2021).

### Choosing and justifying priors

Returning to our political knowledge example, suppose we have good reason to believe (e.g., perhaps thanks to meta‐analysis results) that the regression coefficient for the partial association between age and political knowledge is roughly 0.07 (i.e., for every additional year, we expect political knowledge score to increase by 0.07 points). But, we are only somewhat confident about this specific value; perhaps we would be more comfortable adding some uncertainty such that our prior for age is:

$$b_{\mathrm{age}}∼N\left(0.07,0.1\right),$$

which reads: the regression coefficient of age is normally distributed with a mean of 0.07 and standard deviation of 0.1. Similarly, we can set the prior for education based on previous evidence as:

$$b_{\text{educ}}∼N\left(1.05,0.3\right).$$

These two priors are examples of informative priors because they are informed and justifiable by previous research findings. Because we do not have the same privilege for the intercept and auxiliary (i.e., the standard deviation of the model residuals) parameters, we will use weakly informative, more broad priors to allow a stronger “pull” from the data. These priors will be given by the software default (discussed shortly).

#### Prior predictive checks and sensitivity analysis

Aside from justifying our prior choices, we should conduct prior predictive checks and sensitivity analyses (Depaoli et al., 2020; Gelman et al., 2020; Kruschke, 2021; van de Schoot et al., 2021). Prior predictive checks involve displaying data simulated from the priors' hyperparameters (e.g., mean and variance), which should align with established knowledge or theories (for informative priors) and general expectations (for weakly informative or noninformative priors). The prior predictive distribution, which we simulate, is made from all possible samples that might occur if the model is indeed true. Thus, prior predictive checking involves comparing the observed data with the prior predictive distribution to see if they match up. Ideally, “good” priors will yield prior predictive distributions similar to the real data‐generating distribution (van de Schoot et al., 2021).

Sensitivity analyses consist of reanalyzing the data using different priors to determine the extent to which (and how) our prior choices influence the posterior and consequently, the results. The change in posteriors can often be substantial when using different priors, but this isn't a bad thing. If the sensitivity analysis shows that changes in the prior settings significantly alter our inferences, this is an interesting discovery on its own. This might mean that the theory behind setting the priors has a major influence on the model outcomes, which says something about the (in)stability of the model or theory (Depaoli et al., 2020). On the other hand, if the model results stay reasonably consistent despite changes in prior settings, it implies that the theory (or priors) has less impact on the relationships under investigation. Either way, the results are noteworthy. The bottom line is that researchers need to carefully explore and investigate any interesting changes in the posteriors following adjustments of priors and be honest when sharing their results (Depaoli et al., 2020; Kruschke, 2021).

Prior predictive checks and sensitivity analyses are further demonstrated in the Supporting Information with annotated code, plots, explanations, and suggested readings. It's now time to get our hands dirty with code.

### Bayesian software magic

In this tutorial, we use the **brms** package (Bürkner, 2017), a popular powerhouse designed to fit Bayesian generalised (non‐)linear multivariate multilevel models that facilitates direct interface with Stan (Stan Development Team, 2024). Stan is a powerful statistical programming language geared specifically for performing full Bayesian inference. Both **brms** and Stan are capable of handling advanced statistical models and complex data structures (e.g., multilevel/nested data) and offer extensive options for analysis customization.^2^ Because this tutorial focuses on the basics of Bayesian analysis and inference, it only scratches the surface of software features. We strongly encourage readers to explore the **brms** website and vignettes (paul‐buerkner.github.io/brms).

To install and load the current version of **brms**, we use the following commands:

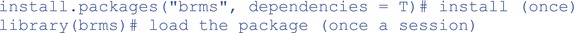

To set the priors we selected for the coefficients of age and education, you can use the set_prior() function:

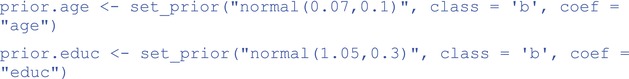

Bear in mind that the first argument in the function specifies the prior distribution family and its hyperparameters; that is, normal (Gaussian) distributions with means of 0.07 and 1.05 and standard deviations of 0.1 and 0.3 for age and education, respectively. The class argument specifies for which type of parameter we are setting the prior. In our case, we set it to “b,” the regression coefficient. If we wanted to specify the priors for the intercept and auxiliary parameters, we could set the class argument to “Intercept” and “sigma,” respectively. To view all the parameters we can specify for this model, we can use the get_prior() function with the formula and data arguments:

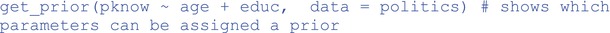

#### Model estimation and posteriors approximation

Now that our priors are defined, we can estimate the model using **brms**'s primary function, brm(). The syntax is similar to that of the lm() function, and it is modelled after the syntax used in the popular frequentist mixed‐effects package, **lme4** (Bates et al., 2015; though, they are not identical).

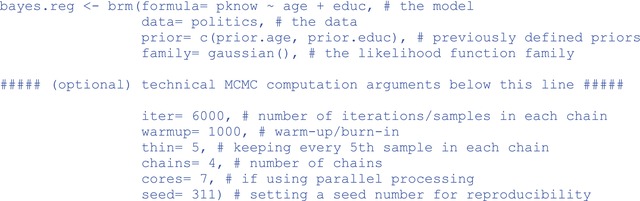

Note the Bayesian‐specific arguments, prior=, which specify the priors, and family=, the distribution type of the likelihood function. Also, keep in mind that there are MCMC specifications already baked into the brm() function by default. Had we not adjusted these manually, the number of chains, the number of iterations in each chain, and the warm‐up period would have been set automatically to chains= 4, iter= 2000, warmup= 1000, and thin= 1, respectively. To extract the results, we can use:

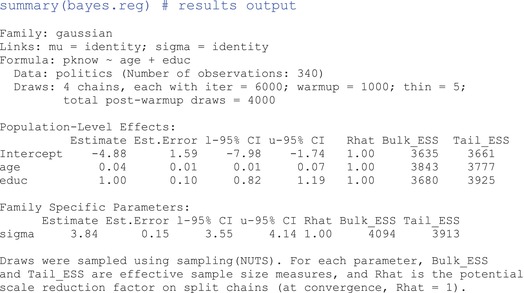

#### Understanding brm() output

We start at the top of the output which shows general information about the model, the data, and MCMC. All of these specifications are customizable as arguments in the brm() function. The Population‐Level Effects section is analogous to the Coefficients section from the lm()output, but includes a few major differences. First, note that there are no significance testing results; there are no *t* or *p* values. Second, each row includes the summary statistics of the approximated posterior probability distribution for a particular model parameter. For example, the approximated posterior distribution of $b_{\text{educ}}$ has a mean of 1.00‐points per year on the political knowledge scale (shown under Estimate) and a standard deviation of 0.10‐points (shown under Est.Error). Note that the default under Estimate is the posterior mean, but you can get the posterior median instead by adding the argument robust= TRUE inside the summary() function. Under l‐95% CI and u‐95% CI are the 2.5th and 97.5th quantiles of the posterior distribution, respectively (the 95% equal‐tailed credible interval), and under Family Specific Parameters are summary statistics depicting the posterior distribution for sigma, the standard deviation of the model residuals (auxiliary parameter).

You might also notice the Rhat, Bulk_ESS, and Tail_ESS columns. These columns refer to $\hat{R}$ and bulk and tail effective sample size (ESS), which are statistics describing the MCMC process. For Bayesian inferences to be valid or hold any merit, the posterior approximation processes that give rise to these inferences must have been properly executed, and it is our job to ensure it did. These statistics are only first‐level checks. In the Supporting Information document, we provide a deeper exploration of MCMC diagnostics, including detailed guidance on plotting the chains. We strongly recommend graphical assessments as a crucial part of the diagnostic process, which we demonstrate in the Supporting Information.

$\hat{R}$ is a convergence diagnostic that compares parameter estimates both between and within chains. Ideally, $\hat{R}$ should be 1. But, if the chains did not mix well (i.e., the between‐ and within‐chain estimates “disagree”), $\hat{R}$ will be greater than 1. It is recommended to run a minimum of four chains and use the parameter estimates only if $\hat{R}$ is less than 1.01 (Vehtari et al., 2021).

Finally, ESS helps to assess the quality and efficiency of the sampling process. Because MCMC uses chained samples, we can expect these to be similar to, or dependent on one another to some degree (recall autocorrelation). But, we still want the samples to be somewhat independent because we need them to explore the parameter space thoroughly and efficiently. ESS is an estimate of the effective number of independent samples drawn from the posterior (either in the bulk of the distribution or the tails), where “the higher the ESS the better,” with a minimum ESS of 400, or 100 per chain (Vehtari et al., 2021, p. 672).

### Bayesian inference: Interpretation and reporting

We can interpret the effects similarly to how we did for the frequentist approach, but we now acknowledge the combination of prior information with the data and the probabilistic nature of the posterior. Referring to $b_{\text{educ}}$, for example, if two random individuals of the same age (i.e., holding age constant) have a one‐year difference in education, based on both prior knowledge and the data, the most likely difference is one point higher on the political knowledge scale for the more educated individual. Aside from the mean of the posterior (or any other measure of centrality), we should also interpret the variability of the posterior or the uncertainty around our most credible parameter value. In fact, it is so important to discuss uncertainty that we dedicate an entire subsection to credible intervals.

#### Credible intervals

Recall the frequentist CI interpretation: if we sample repeatedly from the population and compute CIs for each sample, 95% of CIs will include the population parameter. This definition is quite awkward and cumbersome because it does not include or suggest anything about the interval bounds (or range) we obtained (e.g., [0.01, 0.07] for the effect of age); all we know is that it may or may not include the population parameter value. This definition is also a statement about a long‐term property of our estimation process (i.e., if we repeated this experiment many times). But, we do not repeat the same experiment over and over, we only have one interval in front of us. What we would have liked to obtain from our measure of uncertainty is a range of possible parameter values that have the highest probability of being accurate.

Bayesian statistics use *credible intervals*. Credible intervals are analogous to CIs in the sense that they provide a measure of uncertainty, but they are interpreted differently. As it turns out, the interpretation of credible intervals is much closer to what we would have liked to obtain from a measure of uncertainty; credible intervals present a range of values within which the parameter of interest is believed to have a certain degree of credibility. When we calculate credible intervals, we capture a particular portion (e.g., 95%) of the posterior distribution. This percentage represents the credibility level, and the interval bounds define the range within which we believe the true parameter lies. Credible intervals encapsulate the inherent uncertainty in our best estimate by recognising the variability in the posterior distribution and expressing it as a probabilistic range where the true parameter is most likely to fall. For this reason, credible intervals more directly reflect our uncertainty about our estimates than the frequentist's CI.

There are multiple types of credible intervals, the most common of which are the *Highest Density Interval* (*HDI*) and the *Equal‐Tailed Interval* (*ETI*). HDI involves a process in which we retrieve the narrowest interval that contains the specified probability proportion (e.g., 95%). For a unimodal distribution, this means choosing the values of the highest probability density where each value within the interval has a higher probability than any value outside the interval. ETI, on the other hand, is the interval where the sum of the probabilities below it (i.e., for values smaller than the lower bound) equals the sum of probabilities above it (i.e., for values greater than the upper bound). Put simply, the area under the lower tail should equal the area under the upper tail. If the posterior distribution is symmetric, ETI and HDI produce the same interval.

**Figure 3:**
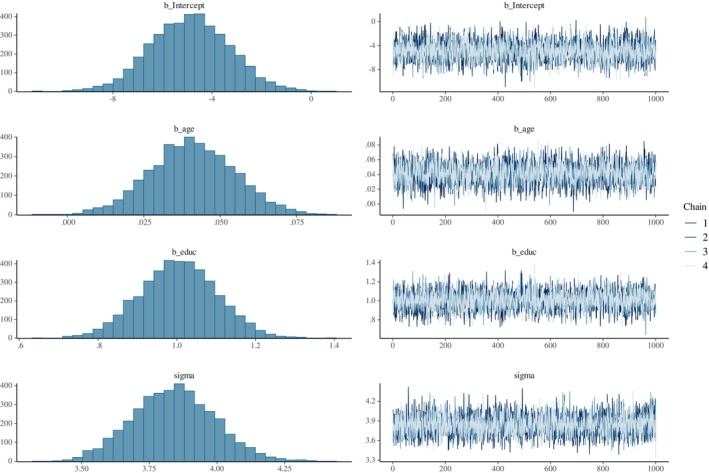
Visualising the posterior distributions and MCMC sampling chains. *Note*: The output from the plot(bayes.reg) function. The four left‐hand side panes show the approximated posterior distributions for the model parameters, whereas the four right‐hand side panes illustrate the post‐warm‐up and thinning MCMC sampling processes. The four chains in each right‐side pane show good “mixture” and converge on a parameter estimate (presented on the *y*‐axis) which represents the centre of its corresponding posterior distribution on the left‐hand side. See the Supporting Information document for more details about interpreting the plots on the right and additional MCMC diagnoses.

The posterior distributions we obtained in our research example are relatively symmetric (see Figure 3), so we should expect similar credible intervals from both HDI and ETI. To retrieve the credible intervals for the regression coefficients in R, we can use functions from **bayestestR** package (Makowski et al., 2019) of the appropriate credible interval type:

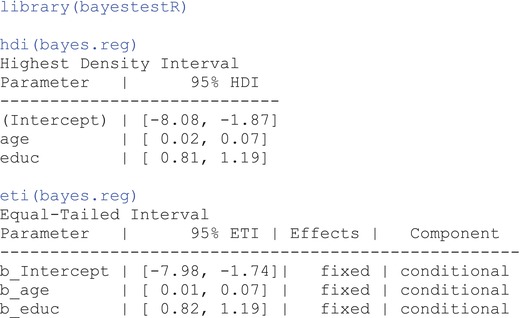

As we expected, HDI and ETI are quite similar. To properly interpret the HDI credible interval associated with the regression coefficient of education, we can state that there is a 95% chance that the population regression coefficient for education is between 0.82 and 1.19 points/year.

#### A note about reporting

When reporting your results, always describe the priors you specified and explain the reasoning behind your selection. Additionally, communicate the posterior distributions for the model parameters with these summary statistics: the value and choice of central tendency measure (e.g., mean or median, for continuous response variables), the probability coverage (e.g., 95%), type (e.g., ETI) and, of course, the bounds of the credible intervals (Kruschke, 2021). Alongside posterior information, researchers should also report on their prior predictive checks, sensitivity analysis, and the computation details such as the software and version used for the analyses, MCMC specifications (e.g., number and length of chains, warm‐up, thinning, etc.) and MCMC diagnostics (see Supporting Information for more details and Kruschke, 2021, for in‐depth best‐practices reporting guidelines). Finally, it is always a good idea to visualise our posterior distributions. The easiest way to visualise the posterior distributions and MCMC chains is with the plot(bayes.reg) function, as shown below. The output is presented in Figure 3. See Supporting Information for more visually appealing and advanced alternatives.

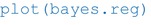

#### *Where did the* p *values go?*

Examining the output from the results summary will reveal no *p* values or any indication of significance testing. Bayesian statistics does not use tests of significance in the traditional sense we know and love (to hate). Instead, the focus is on the magnitude, precision, and probability of the estimated effects. That said, we can still answer questions like “what is the probability that $b_{\text{educ}}=0$?” or, “what is the probability that $b_{\text{educ}}$ is greater than 0.8?” Answers to questions such as these are often what we wish the *p* value would tell us, but it doesn't. To answer these questions, we can calculate the proportion of the posterior that satisfies the condition in question (e.g., $b_{\text{educ}}>0.8$).^3^ With **brms**, we can use the hypothesis() function:

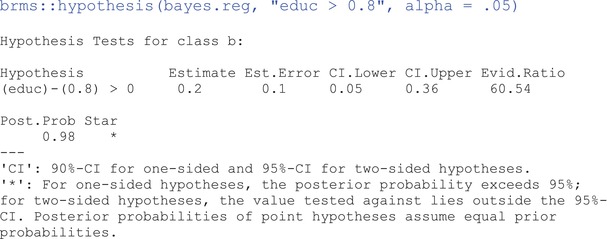

The answer to the last question is found under the last column, Post.Prob. Thus, the estimated probability that $b_{\text{educ}}$ is greater than 0.8 is .98, or 98%. Not surprising, though, considering that the posterior mean is 1. The first four columns in the hypothesis() output describe a distribution representing the difference between the posterior and the hypothesis. Because our $b_{\text{educ}}$ mean is 1, the distribution in the output has a mean of 1–0.8 = 0.2. Evidence ratio (Evid.Ratio) is the ratio of the hypothesis's posterior probability against the posterior of its opposing hypothesis. An evidence ratio greater than 1 indicates stronger evidence for the specified hypothesis by a factor of the ratio value. In contrast, if the ratio is less than 1, the tested hypothesis is less likely than its complement by a factor of 1/ratio. For example, an evidence ratio of .159 would imply that the opposing hypothesis is 1/.159 = 6.23 more likely than the tested hypothesis; or, the specified hypothesis is about 1–.159 = .841, or 84.1% *less* likely than the opposing. In our example, Evid.Ratio is 60.54 which means that $b_{\text{educ}}$ >0.8 is about 60 times more likely than $b_{\text{educ}}$ ≤0.8. Note that for two‐sided hypotheses (e.g., $b_{\text{educ}}=0$), the Evid.Ratio is a Bayes Factor, which is the posterior density at the point of interest divided by the prior density. A brief section on Bayes Factors is found in the Supporting Information.

## CONCLUDING REMARKS

This tutorial provides a gentle introduction to Bayesian statistics tailored for psychology students and researchers using accessible language and applied examples with code. The information provided here is by no means exhaustive, but we believe this paper is a good stepping stone, covering the most relevant concepts and applications for novice Bayesians to embark on this methodological exploration. To help readers take the next step in this exploration, we include a list of recommended readings and resources in the Supporting Information along with additional content and code.

It is important to note that Bayesian statistics has seen significant advancements in instruction on best practices and reporting standards in recent years. Depaoli and van de Schoot (2017) and Kruschke (2021) proposed comprehensive guidelines for conducting and reporting Bayesian analyses in psychological research. These are pivotal contributions to enhancing research findings' validity, reproducibility, and transparency. This tutorial implements many, but not all, of the recommendations proposed in these contributions due to space limitations. We hope that researchers who intend to conduct *any* Bayesian analysis will consult both articles alongside this one.

Our focus on multiple linear regression in this tutorial was intentional, considering its prevalence in psychological research; the general linear model is a familiar and straightforward entry point into more advanced techniques. Further, the foundation built in this paper can be extended to various other analytical methods which go beyond the realm of linear regression. The principles and techniques introduced here can be seamlessly implemented in any type of research context and analytical scenario.

Remember that Bayesian statistics does not negate the frequentist approach. Both perspectives offer valid and effective means of making inferences and drawing conclusions based on observed data. The two statistical schools of thought, however, differ in their underlying perspectives and properties. Embracing Bayesian methods opens a complementary avenue for researchers to broaden their analytical toolkit. Bayesian estimation is a powerful tool that can be applied to any statistical analysis, offering a flexible framework for addressing a diverse range of research questions in psychology.

Indeed, Bayesian estimation is a powerful tool, but it can be computationally heavy and sometimes painfully slow, especially with complex models, due to the iterative process of MCMC. Fortunately, for most of our methodological needs and with a contemporary computing system, the time and computing power it takes to analyse our desired models are usually acceptable and of no major barrier.

One of the advantages of Bayesian statistics is the departure from the traditional emphasis on significance tests. Instead of the limiting dichotomous outcomes of “reject” or “fail to reject,” Bayesian inference encourages a focus on effect size, variability, and probability, helping researchers to be more comfortable with uncertainty. This shift in perspective provides researchers with a more nuanced understanding of the data, fostering a more wholesome interpretation of results that does not confine itself to binary conclusions.

Another noteworthy mention is the importance of priors. Priors lie at the heart of Bayesian thinking and should therefore be an integral part of our analysis plan and execution. Picking priors can sometimes be a daunting and challenging task. Nonetheless, researchers should think carefully about the priors they set without resorting to default or flat priors haphazardly. Because priors can profoundly influence the results, researchers should also avoid setting priors that will help them reach the conclusions they hoped to attain without a proper rationale and justification.

This tutorial was designed as an accessible and fun teaser for the world of Bayesian data analysis. Armed with an understanding of fundamental concepts, terminology, and the spirit of Bayesian statistics we try to impart, we hope readers will feel better prepared to continue their Bayesian education. A deeper understanding of Bayesian logic and skillful practice will not only enhance your statistical analyses but can also lead to more granular and reliable research findings.

## Supporting information


**Data S1.** Additional code, detailed explanations, discussions on further topics, and a curated list of valuable resources.

## Endnotes

^1^
Note that, in practice, there is no need to conduct preliminary analyses using frequentist statistics before delving into Bayesian estimation. The decision to initially use OLS in this paper is deliberate. Its purpose is to establish a shared baseline understanding among all readers and to facilitate a meaningful comparison between frequentist and Bayesian inference methodologies.

^2^
A variety of software platforms support Bayesian analysis, including JAGS, jamovi, JASP, SPSS, Stata and Python. The range and capabilities of software tools are constantly expanding. In R, numerous packages support Bayesian methods; at the time of writing this manuscript, there were at least a few dozen available (see https://cran.r‐project.org/view=Bayesian).

^3^
We use the term “probability” loosely in these questions. What we refer to here is more aligned with the frequentist definition of probability, so we should be careful with interpretations. We should also be cautious not to confuse these results with *p* values or significance testing.
