# Supplementary material for: Navigating the Bayes maze: The psychologist's guide to Bayesian statistics, a hands‐on tutorial with R code
Source: Int J Psychol. 2024 Dec 19;60(1):e13271. doi: 10.1002/ijop.13271 (PMC11658961; doi:10.1002/ijop.13271)
Supplement: Supplementary file 1 — Data S1. Additional code, detailed explanations, discussions on further topics, and a curated list of valuable resources. [file IJOP-60-e13271-s001.html]

Navigating the Bayes Maze: The Psychologist’s Guide to Bayesian Statistics, a Hands-On Tutorial with R Code


# Navigating the Bayes Maze: The Psychologist’s Guide to Bayesian Statistics, a Hands-On Tutorial with R Code

## Supplementary Materials

### Udi Alter, Miranda A. Too, & Robert A. Cribbie

### 2024-13-10

- Getting Set Up
  - Load required packages
- Research
  Example
  - Import data
- Prelude
- Descriptives and data
  visualizations
- Model
  Selection
- Choosing and Justifying
  Priors
- Using
  `brms`
- Prior Predictive Checks
- Model Estimation
  and Posteriors Approximation
- MCMC
  Diagnostics
  - Burn-in
    required
  - Chains converging on
    different values
  - Lack of
    convergence
  - Autocorrelation
  - MCMC
    Statistics
  - What to Do
    When You Encounter Convergence Issues
- Model Fit
- Interpreting `brm()`
  Output
- Plotting the Posterior/s
- Prior Sensitivity Analyses
- Hypothesis
  Testing
  - Bayes Factor
    - Interpreting Bayes Factors
    - Bayes
      Factors in R
- List of Resources and
  Good Reads by Topic
  - Bayesian
    Workflow
  - Textbooks
  - Software
  - Understanding
    Priors and Their Impact (e.g., Sensitivity Analysis, Prior Predictive
    Check)
  - Bayesian Model
    Evaluation and Selection
  - Reporting Guidelines and
    Examples
- Session and Packages
  Information

# Getting Set Up

## Load required packages

```
library(BayesFactor)
library(bayesplot)
library(bayestestR)
library(brms)
library(car)
library(GGally)
library(haven)
library(misty)
library(tidyverse)
```

# Research Example

Suppose a team of researchers is interested in understanding how age
and education affect political knowledge. To answer this question, the
research team surveys \(N = 340\) adult
respondents between the ages of 18 and 90 years residing in the United
States. Participants were asked a series of survey questions about their
knowledge of politics and the political process. Responses from the
survey questions were aggregated into a composite score representing
political knowledge.

## Import data

The data is openly available on Andrew Hayes’s website. You can
download the entire zip file here.
Once you unzip the file and click on the folder labeled “ralm”, look for
the “politics.sav” file inside the POLITICS folder. Make sure that you
save (or move) the “politics.sav” file to your working directory.
Alternatively, you can include the directory path inside the quotation
marks followed by a slash (/) inside the `read_sav()`
function below.

```
politics <-  haven::read_sav("politics.sav") %>% 
  select(pknow, age, educ) # including only variables we will be working with
```

# Prelude

Before we embark on this analysis, we should identify what are the
goals of this analysis. In this example, we intend to:

- Describe the association between education and political
  knowledge in terms of its magnitude and precision
- Explain (as opposed to predict; see Yarkoni & Westfall,
  2017) the nature of the relationship between education and political
  knowledge while partialling out the effects of age, and assessing the
  validity of the linear model with political knowledge regressed on both
  education and age.

We should also report on why we choose a Bayesian approach:

- We would like to incorporate prior knowledge about the
  relationship of interest as reported in previous studies
- Given the somewhat modest sample size, we would like to have
  computationally rigorous, more robust estimates and measures of
  uncertainty

# Descriptives and data visualizations

```
descript(politics)
```

```
##  Descriptive Statistics
## 
##   Variable   n nNA   pNA     M    SD   Min   Max  Skew  Kurt
##    pknow   340   0 0.00% 11.31  4.37  0.00 21.00 -0.09 -0.57
##    age     340   0 0.00% 44.93 14.59 18.00 90.00  0.48 -0.17
##    educ    340   0 0.00% 14.29  2.10  6.00 17.00 -0.58  0.21
```

```
# Function to return points and geom_smooth
# allow for the method to be changed
my_fn <- function(data, mapping, method1="lm", method2= "loess",...){
  p <- ggplot(data = data, mapping = mapping) + 
    geom_point() + 
    geom_smooth(method=method1, size=2)+
    geom_smooth(method=method2, span = 0.7, colour="red", alpha=0.2)
  p
}
# Figure S1 in Supplemental Materials
ggpairs(politics[,c(1,2,3)], aes(alpha = 0.5),
        columnLabels = c("Political knowledge","Age",
                         "Education (years)"),
       # diag=list(continuous=wrap("barDiag", binwidth=7)),
        lower = list(continuous = my_fn),
        upper = list(continuous = wrap('cor', size = 8)))
```

*Note*. The tiles cascading diagonally show the density plot
of the univariate distributions of the individual variables. The tiles
in the upper triangle show the linear correlation coefficients where \*\*\*
indicates statistical significance (p < .001). The tiles in the lower
triangle contain scatterplots representing the bivariate relationship
between each pair of variables. The blue line reflects the fitted slope
of the linear model whereas the red line represents a nonparametric
smoothing using loess with a span of 0.7 to evaluate potential nonlinear
trends.

# Model Selection

To help shed some light on how age and education explain political
knowledge, we can use multiple linear regression with political
knowledge as the outcome variable, whereas age and education are the
regressors. Whether we use frequentist or Bayesian analyses, the linear
model has the same form

\[y\_i = b\_0 + b\_1 X\_{1\_i} + b\_2 X\_{2\_i} +
\epsilon\_i\]

where \(y\_i\) is the *i*th
observed political knowledge value, \(b\_0\) is the population parameter
representing the intercept of the regression plane, \(b\_1\) and \(b\_2\) are the population parameters
representing the partial slopes (regression coefficients) of the first
and second predictor variables, \(X\_{1\_i}\) and \(X\_{2\_i}\) are the \(i\)th (\(i = 1,
…,N\)) observed values for the first and second predictor
variables, and \(\epsilon\_i\) is the
error term, \(\epsilon\_i = y\_i -
\hat{y}\_i\), with \(\hat{y}\_i\)
being the predicted value on the outcome variable for case \(i\). For our research example, we can
rewrite the equation above as

\[pknow\_i = b\_0 + b\_{age}age\_{i} +
b\_{educ} educ\_i\]

where \(pknow\_i\), \(age\_i\), and \(educ\_i\), are the political knowledge, age,
and education values for participant *i*. Descriptive statistics
and data visualizations are presented next.

# Choosing and Justifying Priors

Suppose we have good reason to believe (e.g., perhaps from results of
a meta-analysis) that the regression coefficient for the partial
association between age and political knowledge is roughly 0.07 (i.e.,
for every additional year, we expect political knowledge score to
increase by 0.07-points). But, we are only somewhat confident about this
specific value; perhaps we would be more comfortable adding some
uncertainty such that our prior for age is

\[ {b}\_{age} \sim N(0.07, \
0.01),\]

which reads: the regression coefficient of age is normally
distributed with a mean of 0.07 and variance of 0.01. Similarly, we can
set the prior for education based on previous beliefs or evidence as

\[ {b}\_{education} \sim N(1.05, \
0.09),\]

The two priors we set above are examples of informative priors
because they are informed and justifiable by previous research
findings.

Because we do not have the same privilege for the intercept and
auxiliary parameters (i.e., the standard deviation of the model
residuals), we will use weakly informative, more broad priors to allow a
stronger “pull” from the data. These priors will be given by the
software default.

# Using `brms`

To install and load the current official version of
**brms**, we use the following commands:

```
install.packages("brms", dependencies = T)
```

Next, we load the package so we can use it in this session.

```
library(brms)
```

To set informative priors for the coefficients of age and education,
you can use the `set_prior()` function:

```
prior.age <- set_prior("normal(0.07, 0.1)", class = 'b', coef = "age")

prior.educ <- set_prior("normal(1.05, 0.3)", class = 'b', coef = "educ")
```

Note that the first argument in the function specifies the prior
distribution family and its hyperparameters; that is, a normal
(Gaussian) distribution with a mean of 0.07 and 1.05 and a standard
deviation of 0.1 and 0.3 for age and education, respectively. The class
argument specifies for which type of parameter we are setting the prior.
In our case, we set it to `b`, the regression coefficient. If
we wanted to specify the priors for the intercept and auxiliary
parameters, we could set the class argument to `Intercept`
and `sigma`, respectively. To view all the parameters we can
specify for this model, we can use the `get_prior()` function
with the formula and data arguments:

```
get_prior(pknow ~ age + educ,  data = politics) # shows which parameters can be assigned a prior
```

```
##                  prior     class coef group resp dpar nlpar lb ub       source
##                 (flat)         b                                       default
##                 (flat)         b  age                             (vectorized)
##                 (flat)         b educ                             (vectorized)
##  student_t(3, 11, 4.4) Intercept                                       default
##   student_t(3, 0, 4.4)     sigma                             0         default
```

# Prior Predictive Checks

When doing Bayesian analysis, the accuracy of your results depends a
lot on how good your prior is. It’s really important to check if the
model you’re using actually produces data that makes sense. One way to
do this is called prior predictive checking. Priors are built on what we
already know and, as long as this knowledge is correctly translated into
probabilities, they shouldn’t be wrong. Still, even with a solid method
for creating priors, you need to really understand the specifics of
these probabilities (Daimon, 2008; Moran et al., 2019; van de Schoot et al.,
2021). This is even more crucial for complex models with small
sample sizes because less data means the priors have a bigger impact on
the results (Smid et al.,
2019). Prior predictive checking helps you see how these priors
might affect the observations, but it’s not necessarily about changing
your priors, unless they clearly produce incorrect data (van de Schoot et al.,
2021).

The prior predictive distribution shows all possible samples that
could happen if the model is accurate. Ideally, a “correct” prior will
give a prior predictive distribution similar to the real data-generating
process. Prior predictive checking involves comparing the observed data
(or its statistics, *T*) with the prior predictive distribution
(or its statistics) to see if they match up (van de Schoot et al.,
2021).

To evaluate the prior predictive check, we would need to rerun the
model with the added argument `sample_prior = "only"`, as
seen below

```
# Prior predictive check with the argument sample_prior="only"
prior_pred_check <- brm(formula= pknow ~ age + educ, #the model
                 data=politics, # the data
                     prior = c(prior.age, prior.educ), #previously defined priors
                 family = gaussian(), #the likelihood function family
                 sample_prior = "only") # Simulating prior-based samples
```

```
# Generate prior predictive samples
prior_pred_samples <- posterior_predict(prior_pred_check, 
                                        sample_prior = "only")
attributes(politics$pknow) <- NULL
# Plot the PPC
bayesplot::ppc_stat_2d(y = politics$pknow, yrep = prior_pred_samples) + 
  scale_y_continuous(expand = c(0, 0)) + 
  theme(axis.text = element_text(size = 14),
        axis.title = element_text(size = 16))
```

```
# Generate prior predictive samples
prior_pred_samples <- posterior_predict(prior_pred_check, 
                                        sample_prior = "only")

# Prior kernel density plot
bayesplot::ppc_dens_overlay(y = politics$pknow, 
                                                 yrep = prior_pred_samples[1:40, ], 
                                                 alpha = 1, size = 0.8) + 
  xlim(-500, 500) + 
  ylab("") + 
  theme(axis.text = element_text(size = 14),
        axis.title = element_text(size = 16),
        legend.text = element_text(size = 20))
```

Above, are a few plots comparing the possible samples from the prior
predictive distribution and the observed data. The priors cover the
entire plausible parameter space with the observed data in the centre.
The prior predictive checks for the priors we chose seem reasonable
given the data. But, remember, you might want to repeat this process
with different priors and reassess their appropriateness - this should
be done as part of the sensitivity analysis, which is a crucial step in
Bayesian analyses.

# Model Estimation and Posteriors Approximation

Now that our priors are defined, we can estimate the model using
brms’s primary function, `brm()`. The syntax is similar to
that of the `lm()` function (see previous section), and it is
modeled after the syntax used in the popular frequentist mixed-effects
package, **lme4** (Bates et al.,
2015; though, they are not identical).

```
bayes.reg <- brm(formula= pknow ~ age + educ, #the model
                 data=politics, # the data
                   prior = c(prior.age, prior.educ), #previously defined priors
                 family = gaussian(), #the likelihood function family

##### (optional) technical MCMC computation arguments below this line #####
           
                  iter = 6000, #number of iterations/samples in each chain
                warmup = 1000, #warm-up/burn-in
                thin = 5, # keeping every 5th sample in each chain
                chains = 4, #number of chains
                  cores = 7, #if using parallel processing               
                seed = 311) #setting a seed number for reproducibility
```

Note the new arguments added such as the one specifying the prior/s,
the distribution family of the likelihood function. There are also more
technical specifications relating to the MCMC posterior approximation
process such as the number of chains, the number of samples in each
chain, the warm-up period, the number of cores, and the seed, in case we
want to reproduce these exact results. To extract the results, we can
use

```
summary(bayes.reg) # results output
```

```
##  Family: gaussian 
##   Links: mu = identity; sigma = identity 
## Formula: pknow ~ age + educ 
##    Data: politics (Number of observations: 340) 
##   Draws: 4 chains, each with iter = 6000; warmup = 1000; thin = 5;
##          total post-warmup draws = 4000
## 
## Regression Coefficients:
##           Estimate Est.Error l-95% CI u-95% CI Rhat Bulk_ESS Tail_ESS
## Intercept    -4.88      1.59    -7.98    -1.74 1.00     3635     3661
## age           0.04      0.01     0.01     0.07 1.00     3843     3777
## educ          1.00      0.10     0.82     1.19 1.00     3680     3925
## 
## Further Distributional Parameters:
##       Estimate Est.Error l-95% CI u-95% CI Rhat Bulk_ESS Tail_ESS
## sigma     3.84      0.15     3.55     4.14 1.00     4094     3913
## 
## Draws were sampled using sampling(NUTS). For each parameter, Bulk_ESS
## and Tail_ESS are effective sample size measures, and Rhat is the potential
## scale reduction factor on split chains (at convergence, Rhat = 1).
```

Or, to get only the posteriors’ summaries,

```
posterior_summary(bayes.reg) # with posterior means
```

```
##                  Estimate  Est.Error          Q2.5         Q97.5
## b_Intercept   -4.88146904 1.58997356   -7.97569616   -1.74350944
## b_age          0.04091563 0.01392532    0.01358537    0.06863983
## b_educ         1.00462382 0.09718317    0.81526371    1.19185593
## sigma          3.84040799 0.15106157    3.54911438    4.14419696
## Intercept     11.31102706 0.20696830   10.89779284   11.70990256
## lprior        -3.17678734 0.10596971   -3.45249640   -3.04502642
## lp__        -941.53926031 1.43790954 -945.06336343 -939.76829313
```

To retrieve the credible intervals, use

```
bayestestR::hdi(bayes.reg)
```

```
## Highest Density Interval
## 
## Parameter   |        95% HDI
## ----------------------------
## (Intercept) | [-8.08, -1.87]
## age         | [ 0.02,  0.07]
## educ        | [ 0.81,  1.19]
```

```
bayestestR::eti(bayes.reg)
```

```
## Equal-Tailed Interval
## 
## Parameter   |        95% ETI | Effects |   Component
## ----------------------------------------------------
## b_Intercept | [-7.98, -1.74] |   fixed | conditional
## b_age       | [ 0.01,  0.07] |   fixed | conditional
## b_educ      | [ 0.82,  1.19] |   fixed | conditional
```

# MCMC Diagnostics

Before we celebrate our results, we should also make sure the MCMC
did its job well. Diagnostics of MCMC play a crucial role in assessing
the reliability, validity, and efficiency in approximating the
posterior. There are multiple ways of assessing the MCMC, the most
helpful of which is simply plotting the sample draws and examining the
progression of the iteration process. Using trace plots, we can evaluate
how well the sampling process has explored the parameter space and
converged to the target estimates. There are multiple “red flags” we
should look out for when examining the trace plots. For brevity, we do
not review MCMC diagnostic issues in detail. Nonetheless, we briefly
mention these issues and show how they might appear so that readers can
easily identify a problem should one arise. To get the trace plots, we
can use the following code:

```
bayesplot::mcmc_trace(bayes.reg, pars = vars(b_Intercept, b_age, b_educ, sigma))+theme_minimal()
```

The 5 panes present the entire sampling process of the model
parameters using four chains with 6000 samples each (4000 iterations -
1000 burn-in divided by 5 due to thinning).

We can “zoom-in” on a particular segment from sample such as
iteration 200 to 250:

```
bayesplot::mcmc_trace(bayes.reg, pars = vars(b_Intercept, b_age, b_educ, sigma), window=c(200,250))+theme_minimal()
```

The plots above show the sampling process across all iterations for
each of the model parameters. For each parameter, four chains of 600
samples are used to approximate the posterior. The 1st plot is too busy
and it is difficult to see the individual chains, we can “zoom in” on a
particular segment (e.g., samples 200-250) to get a better view (2nd
plot). Focusing on a small segment, as seen in the 2nd plot, it is
easier to see how the chains “mix” and “dance” around the same parameter
value on the *y*-axis for each of the parameters. When the chains
overlap and interchange (also called “mixture”) as in this case, we can
conclude that the chains are in agreement; they converge on values that
are very similar to one another, which is what we wish to see. We can
also visualize the posteriors generated by each chain overlaying one
another:

```
library(tidybayes)
```

```
## 
## Attaching package: 'tidybayes'
```

```
## The following objects are masked from 'package:brms':
## 
##     dstudent_t, pstudent_t, qstudent_t, rstudent_t
```

```
## The following object is masked from 'package:bayestestR':
## 
##     hdi
```

```
draws <- as_draws_df(bayes.reg) 

draws %>% 
  mutate(chain = .chain) %>% 
  mcmc_dens_overlay(pars = vars(b_Intercept, b_age, b_educ, sigma)) +
  theme_minimal()
```

We can observe close alignment between the 4 chains which increases
our confidence in the reliability and accuracy of the MCMC process.

With a good mixture, we should be able to easily imagine a
horizontal, flat line that crosses in the middle of the chains. Of
course, no single diagnostic or trace plot is perfect, and we can never
be completely certain that the quality of our MCMC sampling process is
sufficient. All we can do is try to detect the presence of issues or red
flags. Here are a few common issues which are also illustrated in plots
below:

## Burn-in required

Burn-in refers to the initial period where the chain has not yet
reached the stationary target. An example is presented at the top-left
panel of Figure S5 showing a short iteration segment of adjustment
before “climbing” to the estimated value and stabilizing.

## Chains converging on different values

We use multiple chains to gain more confidence about the reliability
of our posterior approximation. If one or more chains “disagree” (i.e.,
exhibit diverging patterns) as seen in the top-right corner of Figure
S5, there may be potential problems in convergence and with our
estimates.

## Lack of convergence

When the chain does not stabilize and converge on a particular
target, this might imply that the MCMC process did not explore the
parameter space effectively and a reliable solution was not found. An
example is visible at the bottom-left panel of Figure S5 where the chain
reveals an erratic pattern or a systematic directionality without
settling around a stable location.

## Autocorrelation

Autocorrelation measures the association between a sample and its
lagged versions in the chain. Although MCMC relies on some dependence
between the samples, the issue with high autocorrelation is that it
leads to slower exploration of the parameter space. This is noticeable
in the bottom-right panel in Figure S5 where the waves in the trace plot
have more “momentum” and they change direction less frequently.

We can also plot autocorrelation plots using

```
draws %>% 
  mutate(chain = .chain) %>% 
  mcmc_acf(pars = vars(b_Intercept, b_age, b_educ, sigma), lags = 35) +
  theme_minimal()
```

```
summary(bayes.reg)
```

```
##  Family: gaussian 
##   Links: mu = identity; sigma = identity 
## Formula: pknow ~ age + educ 
##    Data: politics (Number of observations: 340) 
##   Draws: 4 chains, each with iter = 6000; warmup = 1000; thin = 5;
##          total post-warmup draws = 4000
## 
## Regression Coefficients:
##           Estimate Est.Error l-95% CI u-95% CI Rhat Bulk_ESS Tail_ESS
## Intercept    -4.88      1.59    -7.98    -1.74 1.00     3635     3661
## age           0.04      0.01     0.01     0.07 1.00     3843     3777
## educ          1.00      0.10     0.82     1.19 1.00     3680     3925
## 
## Further Distributional Parameters:
##       Estimate Est.Error l-95% CI u-95% CI Rhat Bulk_ESS Tail_ESS
## sigma     3.84      0.15     3.55     4.14 1.00     4094     3913
## 
## Draws were sampled using sampling(NUTS). For each parameter, Bulk_ESS
## and Tail_ESS are effective sample size measures, and Rhat is the potential
## scale reduction factor on split chains (at convergence, Rhat = 1).
```

Identifying these or related issues in the trace plots can help
researchers detect potential problems with the MCMC sampling process and
take corrective actions (e.g., removing the burn-in period, thinning,
selecting a different starting value, etc.). Remember that it is always
a good idea to use multiple diagnostics and conduct a thorough
assessment of the MCMC results to increase the chances of a valid and
reliable Bayesian inference.

## MCMC Statistics

We can also evaluate our MCMC process using MCMC statistics which are
found in the result summary, under the
`Regression Coefficients:` section. Specifically, we are
looking for effective sample size (`bulk_ESS` and
`tail_ESS`) and \(\hat{R}\)
(`Rhat`).

```
summary(bayes.reg)
```

```
##  Family: gaussian 
##   Links: mu = identity; sigma = identity 
## Formula: pknow ~ age + educ 
##    Data: politics (Number of observations: 340) 
##   Draws: 4 chains, each with iter = 6000; warmup = 1000; thin = 5;
##          total post-warmup draws = 4000
## 
## Regression Coefficients:
##           Estimate Est.Error l-95% CI u-95% CI Rhat Bulk_ESS Tail_ESS
## Intercept    -4.88      1.59    -7.98    -1.74 1.00     3635     3661
## age           0.04      0.01     0.01     0.07 1.00     3843     3777
## educ          1.00      0.10     0.82     1.19 1.00     3680     3925
## 
## Further Distributional Parameters:
##       Estimate Est.Error l-95% CI u-95% CI Rhat Bulk_ESS Tail_ESS
## sigma     3.84      0.15     3.55     4.14 1.00     4094     3913
## 
## Draws were sampled using sampling(NUTS). For each parameter, Bulk_ESS
## and Tail_ESS are effective sample size measures, and Rhat is the potential
## scale reduction factor on split chains (at convergence, Rhat = 1).
```

`Rhat`, `Bulk_ESS`, and `Tail_ESS`
refer to \(\hat{R}\), bulk, and tail
effective sample size (ESS), which are statistics describing the MCMC
process. For Bayesian inferences to be valid or hold any merit, the
posterior approximation processes that give rise to these inferences
must have been properly executed, and it’s our job to ensure it did.
These statistics are only first-level checks.

\(\hat{R}\) is a convergence
diagnostic that compares parameter estimates both between and within
chains. It is the ratio of the average variance of samples within each
chain to the variance of all samples across all chains. Ideally, \(\hat{R}\) should be 1. But, if the chains
did not mix well (i.e., the between- and within-chain estimates
disagree), \(\hat{R}\) will be greater
than 1. It is recommended to run a minimum of four chains and use the
parameter estimates only if \(\hat{R}\)
is less than 1.01 (Vehtari
et al., 2021).

Finally, ESS helps to assess the quality and efficiency of the
sampling process. Because MCMC uses chained samples, we can expect these
to be similar to, or dependent on one another to some degree
(autocorrelation). But, we still want the samples to be somewhat
independent because we need them to explore the parameter space
thoroughly and efficiently. ESS is an estimate of the effective number
of independent samples drawn from the posterior (either in the bulk of
the distribution or the tails), where “the higher the ESS the better,”
with a minimum ESS of 400, or 100 per chain (Vehtari
et al., 2021, p. 672).

ESS divides the sample size (in our case, \(N=340\)) by the amount of autocorrelation.
It is an exchange rate of sorts between the dependent and independent
sample draws in the MCMC iterative process. If there is high
autocorrelation, the ESS value will be smaller. Ideally, we hope for
values much greater than the number of iterations.

ESS required for an accurate and stable representation of the
posterior distribution depends on the specific details you want to
capture. For features influenced by dense regions in the parameter
space, such as the median in unimodal distributions, a smaller ESS will
likely be sufficient. However, for features influenced by sparse
regions, like the 95% Highest Density Interval (HDI) limits, a larger
ESS is necessary. This is because sparse regions are less frequently
sampled, requiring longer chains to get a high-resolution view.

## What to Do When You Encounter Convergence Issues

Convergence issues in Bayesian analysis arise when the MCMC algorithm
struggles to explore the parameter space effectively and find a
solution, leading to unreliable posterior estimates. Like any other
issue (not just in statistics), convergence issues can stem from a
variety of causes. While it’s impossible to address all potential
scenarios here, it’s important to start by diagnosing the problem. Using
MCMC diagnostics, trace plots, and statistical checks can help identify
the root cause for the convergence issues. With this understanding, here
are some common steps that may help address these problems:

- **Increase the number of iterations and chains**:
  Sometimes, MCMC chains require more samples to adequately explore the
  parameter space. Increasing the number of iterations or the number of
  chains (e.g., from 4 to 6) can help the sampler converge on the
  posterior distribution. In the `brm()` function, you can
  increase the number of iteration by adjusting the value corresponding to
  the `iter =` argument. Similarly, the number of chains can be
  adjusted with the `chains =` argument.
- **Adjust the warm-up/burn-in period**: The warm-up
  or burn-in period allows the MCMC to stabilize before collecting
  samples. If chains fail to converge, increasing the burn-in period gives
  them more time to adjust to the target distribution. Users can use the
  `warmup =` argument in the `brm()`
  function.
- **Consider adjusting priors**: Strongly informative
  priors can sometimes pull the posterior too forcefully toward a specific
  region, leading to convergence issues. Increasing the uncertainty or
  spread of the informative prior, trying a weakly informative priors, or
  re-evaluating the appropriateness of the chosen priors can mitigate this
  issue.
- **Thin the chains**: Thinning involves keeping only
  every nth sample (e.g., every 5th) to reduce autocorrelation between
  samples, making the chains more independent and improving the chances of
  convergence success. Adjusting the thinning can be done using the
  `thin =` argument.
- **Consider the appropriateness of the model**:
  Sometimes convergence issues arise because the model itself may be too
  complex or poorly specified. It’s important to evaluate whether the
  model structure is appropriate for the data and the research question.
  Simplifying the model, removing unnecessary parameters, or ensuring the
  model aligns with the underlying data-generating process can often
  improve convergence. For instance, check whether all predictors are
  necessary or if there are redundant or collinear variables that could be
  removed.
- **Assess trace plots and R-hat values**: Examining
  trace plots for “mixing” of the chains and computing the R-hat
  diagnostic (which should be close to 1) will help detect whether the
  chains have properly converged. If not, consider rerunning the model
  with more robust computational settings (e.g., more iterations, fewer
  thinned samples).
- **Use alternative samplers**: Some issues are
  specific to the sampling algorithm used. For example, switching from the
  default NUTS (No-U-Turn Sampler) to another algorithm like Hamiltonian
  Monte Carlo (HMC) in `brms` can help address convergence
  issues.

Although this list of suggestions is helpful, it is not exhaustive
and doesn’t go in-depth, given that this is an introductory tutorial.
It’s important to approach each issue systematically, using diagnostics
and trial-and-error, while considering the specific characteristics of
your analysis. Additionally, any convergence problems and the steps
taken to address them should be reported transparently in your results.
This includes detailing any adjustments made and presenting sensitivity
analyses to demonstrate the robustness of your findings under different
model settings. Transparency in handling these issues is crucial for
ensuring the credibility and reproducibility of the analysis.

# Model Fit

With OLS, we use multiple \(R^2\) to
assess the fit of the model. In Bayesian statistics, we have the
**posterior predictive distribution (PPD)**. We use the PPD
to evaluate how well the model fits the data. The idea behind assessing
the adequacy of the model using the PPD is simple: Using the resulting
posterior distribution(s) to inform our model about the parameters of
interest, a new, hypothetical dataset is generated (predicted). If the
model we estimated is adequate, then any fictitious data we generate
using this model (i.e., model-implied predictions) should resemble our
observed data (e.g., political knowledge scores). We can compare the
distribution of the outcome variable generated from the model with the
actual observed data to assess whether the model sufficiently captures
the patterns and variability found in the observed data. Deviations
between the observed and predicted data might suggest potential
limitations in our model specification and can guide model refinement.
Thus, the PPD indicates the likelihood of future observations
(predictions) given the estimated parameters, the data, and our
model.

A good approach to assess PPD is to plot the observed outcome
distribution against the PPD which we can do using the code below:

```
pp_check(bayes.reg, ndraws = 1000)+theme_minimal()
```

The posterior predictive distribution is in light blue whereas the
observed pknow scores are in dark blue. Because the two distributions
seem well-aligned, we can conclude that the model fits sufficiently
well.

There are a lot of other interesting ways to visualize model fit
using PPD, for example.

# Interpreting `brm()` Output

Let’s look at the results, again:

```
summary(bayes.reg) # results output
```

```
##  Family: gaussian 
##   Links: mu = identity; sigma = identity 
## Formula: pknow ~ age + educ 
##    Data: politics (Number of observations: 340) 
##   Draws: 4 chains, each with iter = 6000; warmup = 1000; thin = 5;
##          total post-warmup draws = 4000
## 
## Regression Coefficients:
##           Estimate Est.Error l-95% CI u-95% CI Rhat Bulk_ESS Tail_ESS
## Intercept    -4.88      1.59    -7.98    -1.74 1.00     3635     3661
## age           0.04      0.01     0.01     0.07 1.00     3843     3777
## educ          1.00      0.10     0.82     1.19 1.00     3680     3925
## 
## Further Distributional Parameters:
##       Estimate Est.Error l-95% CI u-95% CI Rhat Bulk_ESS Tail_ESS
## sigma     3.84      0.15     3.55     4.14 1.00     4094     3913
## 
## Draws were sampled using sampling(NUTS). For each parameter, Bulk_ESS
## and Tail_ESS are effective sample size measures, and Rhat is the potential
## scale reduction factor on split chains (at convergence, Rhat = 1).
```

We start at the top of the output which shows general information
about the model, the data, and MCMC. All of these specifications are
customizable as arguments in the `brm()` function. Right
under, is the `Population-Level Effects` section which is
analogous to the `Coefficients` section from the
`lm()` summary output, but includes a few major
differences.

First, note that there are no significance testing results; there are
no *t* or *p* values. Second, each row includes the
summary statistics of the estimated posterior probability distribution
for a particular model parameter. For example, the posterior
distribution of \(b\_{educ}\) has a mean
of 1.00-points per year on the political knowledge scale (shown under
`Estimate`) and a standard deviation of 0.10-points (shown
under `Est.Error`). Note that the **brms**
default under `Estimate` is the posterior mean, but you can
get the posterior median instead by adding the argument
`robust=TRUE` inside the `summary()` function. For
example:

```
posterior_summary(bayes.reg, robust = TRUE) # # with median and the median absolute deviation (MAD)
```

```
##                 Estimate  Est.Error          Q2.5         Q97.5
## b_Intercept   -4.8802124 1.59642704   -7.97569616   -1.74350944
## b_age          0.0407869 0.01374194    0.01358537    0.06863983
## b_educ         1.0049992 0.09675354    0.81526371    1.19185593
## sigma          3.8399921 0.15258468    3.54911438    4.14419696
## Intercept     11.3125772 0.20528416   10.89779284   11.70990256
## lprior        -3.1534962 0.08144832   -3.45249640   -3.04502642
## lp__        -941.2079463 1.24621820 -945.06336343 -939.76829313
```

Under `l-95% CI` and `u-95% CI`, are the 2.5th
and 97.5th quantiles of the posterior distribution, respectively (the
95% equal-tailed credible interval), and under
`Family Specific Parameters` are summary statistics depicting
the posterior distribution for sigma, the standard deviation of the
model residuals or auxiliary parameter.

# Plotting the Posterior/s

The easiest way to visualize the posterior distributions and MCMC
chains is with the `plot()` and `pairs()`
functions.

```
plot(bayes.reg)
```

```
pairs(bayes.reg,
      off_diag_args = list(size = 1/3, alpha = 1/3))
```

The next plot shows the posterior distribution of education,
highlighting its mean and 95% credible interval.

```
library(bayesplot)
mcmc_areas(bayes.reg, pars=c("b_educ"), prob=.95)+
  theme_minimal()
```

```
mcmc_areas(bayes.reg, pars=c("b_age"), prob=.95)+
  theme_minimal()
```

```
library(tidybayes)

draws %>% 
  ggplot(aes(x = b_educ, y = 0)) +
  stat_halfeye(point_interval = mean_hdi, .width = .95) +
  scale_y_continuous(NULL, breaks = NULL)
```

```
draws %>% 
  ggplot(aes(x = b_age, y = 0)) +
  stat_halfeye(point_interval = mean_hdi, .width = .95) +
  scale_y_continuous(NULL, breaks = NULL)
```

Another useful way of visualizing the posterior distribution is by
plotting the estimated regression lines as illustrated below: We
randomly sampled 1000 regression coefficients that correspond to
education (of the same model we used above) from the approximated
posterior distribution. These 1000 slopes are presented in light grey
whereas their mean is in blue. Because we have 1000 regression lines, it
looks like a grey band. But, these are just many light grey lines. The
code to reproduce this plot is at the end of this document.

```
fits <- draws %>%
  as_tibble() %>%
  rename(intercept = `b_Intercept`, educ = b_educ) %>%
  select(-sigma)

#head(fits)
# aesthetic controllers
n_draws <- 1000
alpha_level <- .15
color_draw <- "grey60"
color_mean <-  "#3366FF"
# make the plot
ggplot(politics) +
  # first - set up the chart axes from original data
  aes(x = educ, y = pknow ) +
  # restrict the y axis to focus on the differing slopes in the
  # center of the data
  coord_cartesian(ylim = c(0, 21)) +
  # Plot a random sample of rows from the simulation df
  # as gray semi-transparent lines
  geom_abline(
    aes(intercept = intercept, slope = educ),
    data = sample_n(fits, n_draws),
    color = color_draw,
    alpha = alpha_level
  ) +
  # Plot the mean values of our parameters in blue
  # this corresponds to the coefficients returned by our
  # model summary
  geom_abline(
    intercept = mean(fits$intercept),
    slope = mean(fits$educ),
    size = 1,
    color = color_mean
  ) +
  geom_jitter() +
  # set the axis labels and plot title
  labs(x = 'Education (years)',
    y = 'Political knowledge' ,
    title = 'Visualization of Regression Lines From the Posterior Distribution')
```

```
my_breaks <-
  mode_hdi(draws$b_educ)[, 1:3] %>% 
  pivot_longer(everything(), values_to = "breaks") %>% 
  mutate(labels = breaks %>% round(digits = 3))

draws %>% 
  ggplot(aes(x = b_educ, y = 0)) +
  stat_histinterval(point_interval = mode_hdi, .width = .95,
                    fill = "darkviolet", slab_color = "white",
                    breaks = 40, slab_size = .25, outline_bars = T) +
  scale_x_continuous(breaks = my_breaks$breaks,
                     labels = my_breaks$labels) +
  scale_y_continuous(NULL, breaks = NULL) +
  labs(x = "Education regression coefficient") +
  theme_minimal()
```

We can also visualize the posterior of education generated by each
chain:

```
draws %>% 
  mutate(chain = .chain) %>% 
  mcmc_dens_overlay(pars = vars(b_educ))
```

Here, we see that the four chains are quite consistent and are
overlying closely. This is something we discussed in MCMC diagnostics,
too.

# Prior Sensitivity Analyses

Sensitivity analysis is essential for comprehending Bayesian results
in applied research. Without conducting a sensitivity analysis, it’s
challenging to distinguish the prior’s impact from the data’s impact
during the estimation process; a sensitivity analysis simply helps
researchers to understand the relative influence of priors versus data
(Depaoli
et al., 2020).

In sensitivity analyses, different priors are entertained and we can
use Bayes factors to compare the same model using different priors so we
can assess which priors are best supported by the data.

Recall the priors we set for our primary model,
`bayes.reg`

```
prior_summary(bayes.reg)
```

```
##                  prior     class coef group resp dpar nlpar lb ub  source
##                 (flat)         b                                  default
##      normal(0.07, 0.1)         b  age                                user
##      normal(1.05, 0.3)         b educ                                user
##  student_t(3, 11, 4.4) Intercept                                  default
##   student_t(3, 0, 4.4)     sigma                             0    default
```

Now, let’s try some other priors for education and see how the
posterior distributions change.

```
prior.educ2 <- set_prior("normal(0.75, sqrt(0.0625))", class = 'b', coef = "educ")
prior.educ3 <- set_prior("normal(0, 1)", class = 'b', coef = "educ")
prior.educ4 <- set_prior("normal(3, 1)", class = 'b', coef = "educ")
```

Now, let’s rerun the same model, using the different priors:

```
bayes.reg2 <- brm(formula= pknow ~ age + educ, #the model
                 data=politics, # the data
                     prior = c(prior.age, prior.educ2), #previously defined priors
                 family = gaussian(), #the likelihood function family

##### (optional) technical MCMC computation arguments below this line #####
           
                    iter = 6000, #number of iterations/samples in each chain
                warmup = 1000, #warm-up/burn-in
                thin = 5, # keeping every 5th sample in each chain
                chains = 4, #number of chains
                    cores = 7, #If using parallel processing               
                seed = 311) #setting a seed number for reproducibility


bayes.reg3 <- brm(formula= pknow ~ age + educ, #the model
                 data=politics, # the data
                     prior = c(prior.age, prior.educ3), #previously defined priors
                 family = gaussian(), #the likelihood function family

##### (optional) technical MCMC computation arguments below this line #####
           
                    iter = 6000, #number of iterations/samples in each chain
                warmup = 1000, #warm-up/burn-in
                thin = 5, # keeping every 5th sample in each chain
                chains = 4, #number of chains
                    cores = 7, #If using parallel processing               
                seed = 311) #setting a seed number for reproducibility

bayes.reg4 <- brm(formula= pknow ~ age + educ, #the model
                 data=politics, # the data
                     prior = c(prior.age, prior.educ4), #previously defined priors
                 family = gaussian(), #the likelihood function family

##### (optional) technical MCMC computation arguments below this line #####
           
                    iter = 6000, #number of iterations/samples in each chain
                warmup = 1000, #warm-up/burn-in
                thin = 5, # keeping every 5th sample in each chain
                chains = 4, #number of chains
                    cores = 7, #If using parallel processing               
                seed = 311) #setting a seed number for reproducibility
```

Next, we’re going to plot the different \(b\_{educ}\) posteriors corresponding to the
different priors.

```
# we first sample from the different posterior
original_posterior <- posterior_samples(bayes.reg, pars = "b_educ")
posterior_2 <- posterior_samples(bayes.reg2, pars = "b_educ")
posterior_3 <- posterior_samples(bayes.reg3, pars = "b_educ")
posterior_4 <- posterior_samples(bayes.reg4, pars = "b_educ")

# Then, combine them all to one dataframe for easier plotting
posteriors1234 <- bind_rows("prior.educ" = gather(original_posterior),
                            "prior.educ2" = gather(posterior_2),
                            "prior.educ3" = gather(posterior_3),
                            "prior.educ4" = gather(posterior_4),
                            .id = "Prior")
# changing the column names for convenience
colnames(posteriors1234) <- c("Prior" ,"Posterior"  , "value")

posterior_stats <- posteriors1234 %>%
  group_by(Prior) %>%
  summarize(median_value = median(value),
            lower_ci = quantile(value, 0.05),
            upper_ci = quantile(value, 0.95),
            mean_ci = mean(c(quantile(value, 0.05), quantile(value, 0.95))))

# Calculate density data for each group
density_data <- posteriors1234 %>%
  group_by(Prior) %>%
  do(data.frame(density(.$value)[c("x", "y")]))

# Extract the max density values
max_density_values <- density_data %>%
  group_by(Prior) %>%
  summarize(max_density = max(y))

# Combine stats and max density values
posterior_stats <- left_join(posterior_stats, max_density_values, by = "Prior")

# Define transparency levels
alpha_levels <- c("prior.educ" = 1, "prior.educ2" = 0.3, "prior.educ3" = 0.3, "prior.educ4" = 0.3)

# Define height for credible intervals
ci_height <- c(0.1, 0.2, 0.3, 0.4)  # Fixed height for credible interval lines

# Plotting
ggplot(data = posteriors1234, 
       mapping = aes(x = value, colour = Prior, linetype = Prior)) +
  geom_density(size = 1.2) +
  theme_minimal() +
  geom_segment(data = posterior_stats, 
               aes(x = lower_ci, xend = upper_ci, y = ci_height, yend = ci_height, 
                   colour = Prior, linetype = Prior),
               size = 1) +
  geom_point(data = posterior_stats, 
             aes(x = mean_ci, y = ci_height, 
                 colour = Prior, fill = Prior),
             size = 3, shape = 21) +
  geom_segment(data = posterior_stats, 
               aes(x = lower_ci, xend = lower_ci, y = ci_height - 0.01, yend = ci_height + 0.01, 
                   colour = Prior, linetype = Prior),
               size = 1) +
  geom_segment(data = posterior_stats, 
               aes(x = upper_ci, xend = upper_ci, y = ci_height - 0.01, yend = ci_height + 0.01, 
                   colour = Prior, linetype = Prior),
               size = 1)+xlab("Parameter Value")+ylab("Density")
```

In the plot above, we see each of the posterior distributions for
\(b\_{educ}\) which correspond to the
different priors. Each posterior has a different line type and colour.
We can also see the 90% credible interval associated with each posterior
and its mean illustrated by the coloured circle in the middle of the
interval.

Despite some subtle variations between the distributions, means, and
credible intervals - the distributions and credible intervals overlap
substantially - the different priors we set for \(b\_{educ}\) do not influence our posterior,
and thus our results, to a meaningful degree. This gives us further
support for the robustness of our inference and confidence in the prior
we originally set for \(b\_{educ}\).

In this scenario we see very little change in the results when using
different priors. However, the change in posteriors can often be
substantial when using different priors, and it might feel intimidating
at first to see that the sensitivity analysis shows priors strongly
affecting the final model estimates. But this isn’t something to worry
about too much. If the sensitivity analysis shows that even small
changes in the prior settings significantly alter the final results,
it’s an important discovery, too. This might mean that the theory behind
setting the priors has a major influence on the model outcomes, and
discovering this helps us understand how stable the model or theory is.
On the other hand, if the model results stay pretty consistent despite
changes in prior settings, it shows that the theory (or priors) has less
impact on the findings. Either way, the results are noteworthy and
should be thoroughly explained in the discussion. Understanding how
priors influence the model will ultimately lead to more refined and
informed theories in the field Depaoli
et al., 2020. The bottom line is that the researcher needs to
carefully explore and investigate any interesting changes in the
posteriors following adjustments of priors and be honest when sharing
their results (Kruschke,
2021).

Note that for this demonstration, we only perform prior sensitivity
analysis for a single parameter, \(b\_{educ}\). However, the prior sensitivity
analysis should be done for all parameters.

# Hypothesis Testing

Examining the output from the results summary will reveal no
*p* values or any indication of significance testing. Bayesian
statistics does not use tests of significance in the traditional sense
we know and love (to hate). Instead, the focus is on the magnitude,
precision, and probability of the estimated effects. That said, we can
still answer questions like “what is the probability that \(b\_{educ}=0\)?” or, “what is the probability
that \(b\_{educ}\) is greater than 0.8?”
Answers to questions such as these are often what we wish the *p*
value would tell us, but it doesn’t. To answer these questions, we can
calculate the proportion of the posterior that satisfies the condition
in question (e.g., \(b\_{educ}>0.8\)). With
**brms**, we can use the `hypothesis()`
function:

```
brms::hypothesis(bayes.reg, "educ > 0.8", alpha = .05)
```

```
## Hypothesis Tests for class b:
##         Hypothesis Estimate Est.Error CI.Lower CI.Upper Evid.Ratio Post.Prob
## 1 (educ)-(0.8) > 0      0.2       0.1     0.05     0.36      60.54      0.98
##   Star
## 1    *
## ---
## 'CI': 90%-CI for one-sided and 95%-CI for two-sided hypotheses.
## '*': For one-sided hypotheses, the posterior probability exceeds 95%;
## for two-sided hypotheses, the value tested against lies outside the 95%-CI.
## Posterior probabilities of point hypotheses assume equal prior probabilities.
```

The answer to the last question is found under the last column,
`Post.Prob`. Thus, the estimated probability that \(b\_{educ}\) is greater than 0.8 is 0.98, or
98%. Not surprising, though, considering that the posterior mean is 1.
The first four columns in the `hypothesis()` output describe
a distribution representing the difference between the posterior and the
hypothesis. Because our \(b\_{educ}\)
mean is 1, the distribution in the output has a mean of 1-0.8=2.
Evidence ratio (`Evid.Ratio`) is the ratio of the
hypothesis’s posterior probability against its opposing hypothesis. An
evidence ratio greater than 1 indicates stronger evidence for the
specified hypothesis by a factor of the ratio value. In contrast, if the
ratio is less than 1, the tested hypothesis is less likely than its
complement by a factor of 1/ratio. For example, an evidence ratio of
0.159 would imply that the opposing hypothesis is 1/0.159=6.23 more
likely than the tested hypothesis; or, the specified hypothesis is about
84.1% less likely than the opposing.

In our example, `Evid.Ratio` is 60.54 which means that
\(b\_{educ}>0.8\) is about 60 times
more likely than \(b\_{educ}<0.8\).
Note that for two-sided hypotheses (e.g., \(b\_{educ}=0\)), the `Evid.Ratio`
is a **Bayes factor**, which is the posterior density at
the point of interest divided by the prior density.

## Bayes Factor

A Bayes Factor (BF) is a statistic used to compare two competing
hypotheses or models. BF measures the strength of evidence in favour of
one hypothesis over the other, given the observed data. It’s a ratio
that quantifies how much more likely the observed data is under one
model compared to another.

> “The factor by which the data shift one’s prior beliefs about the
> relative plausibility of two competing models is now widely known as the
> Bayes factor, and it is arguably the gold standard for Bayesian model
> comparison and hypothesis testing.”
>
> — Verhagen & Wagenmakers (2016, p.19)

Let’s break it down a bit further. We start with some terms and their
definitions:

- Prior Odds: The ratio of the probabilities of the two models
  (e.g., Model 1 and Model 0; M1 and M0) before looking at the data (e.g.,
  \(\frac{P(M1)}{P(M0)}\))
- Posterior Odds: the ratio of the probabilities of the two models
  after considering the data (e.g., \(\frac{P(M1|D)}{P(M0|D)}\))
- Bayes Factor: It is the ratio of Posterior Odds to Prior Odds, or
  the factor by which the Prior Odds are multiplied to obtain the
  Posterior Odds. It tells us how the data has changed our belief in the
  models

\[BF\_{M1/M0}=\frac{P(M1|D)}{P(M0|D)}/\frac{P(M1)}{P(M0)}\]

After some rearrangements and simplifications using Bayes’s Theorem,
we can rewrite this equation as

\[BF\_{M1/M0}=\frac{P(D|M1)}{P(D|M0)}\]

Note the location “switch” between the model and D inside the
brackets. Now, we’re dealing with P(D|M), which is the likelihood of the
data, given the model (or hypothesis). Thus, what we see is that BF is
the ratio of the likelihood of the data under M1 to the likelihood of
the data under M0. Cool, right?

### Interpreting Bayes Factors

Generally, if the BF value is larger than 1, we say that the observed
data are more likely under M1 than under M0. If BF is smaller than 1, it
is the other way around; BF < 1 implies that the observed data are
more likely under M0 than under M1. If BF is 1, the data are as likely
under M1 as they are under M0.

### Bayes Factors in R

Here’s an example. Let’s evaluate our model, `bayes.reg`,
against one of the alternative models generated as part of the
sensitivity analysis discussed earlier. You’ll need the
**BayesFactor** package

```
library(BayesFactor)
bf <- bayes_factor(bayes.reg, bayes.reg2)
```

```
bf
```

```
## Estimated Bayes factor in favor of bayes.reg over bayes.reg2: 1.29671
```

Here, we see that BF=1.297 which implies that, given the observed
data, the `bayes.reg` model (with the prior for \({b}\_{education} \sim N(1.05, \ 0.09)\)) is
1.29 times more likely than the `bayes.reg2` model which has
the \({b}\_{education} \sim N(0.75, \
0.0625)\) prior.

Right above, we actually compared the priors using BF, because the
model was the same in terms of its variables and parameters. But, you
can also compare models containing different variables or parameters,
for example, perhaps you’d want to check whether a model
*without* the predictor of age is more likely. You can define
that model such that

```
bayes.reg.educ.only <- brm(formula= pknow ~ educ, #the model
                 data=politics, # the data
                     prior = prior.educ, #previously defined priors
                 family = gaussian(), #the likelihood function family

##### (optional) technical MCMC computation arguments below this line #####
           
                    iter = 6000, #number of iterations/samples in each chain
                warmup = 1000, #warm-up/burn-in
                thin = 5, # keeping every 5th sample in each chain
                chains = 4, #number of chains
                    cores = 7, #If using parallel processing               
                seed = 311) #setting a seed number for reproducibility


bfage <- bayes_factor(bayes.reg.educ.only, bayes.reg)
```

```
bfage
```

```
## Estimated Bayes factor in favor of bayes.reg.educ.only over bayes.reg: 0.13675
```

BF=0.137 which implies that, given the observed data, the
`bayes.reg.educ.only` model (having only the predictor of
education, without age) is 0.14 times more likely than the
`bayes.reg` model. Note, that BF is less than 1 which implies
that `bayes.reg.educ.only` is **not** more
likely. In fact, this hypothesized model without `age` is
\((1-BF) \cdot 100\% = (1- 0.14) \cdot 100\% =
86\%\) **less** likely that our original model with
includes both education and age as predictors. Or, alternatively, you
can say that our original model, `bayes.reg` is \(\frac{1}{BF}=\frac{1}{0.14}=7.3\) times
more likely than the `bayes.reg.educ.only` model. This would
be identical to changing the order of appearance of the models in the
`bayes_factor()` function. Take a look:

```
bayes_factor(bayes.reg, bayes.reg.educ.only)
```

```
## Iteration: 1
## Iteration: 2
## Iteration: 3
## Iteration: 4
## Iteration: 5
## Iteration: 1
## Iteration: 2
## Iteration: 3
## Iteration: 4
```

```
## Estimated Bayes factor in favor of bayes.reg over bayes.reg.educ.only: 7.29823
```

# List of Resources and Good Reads by Topic

Here, we provide just a few good reads and resources. But, there are
plenty more out there, most of which will be made easier to read and
much more accessible to novice Bayesians after reading the tutorial to
which this document is accompanied.

## Bayesian Workflow

- Bayesian
  Workflow (Gelman et al., 2020)
- Bayesian
  Analysis Reporting Guidelines (Kruschke, 2021)
- Bayesian
  statistics and modelling (van de Schoot et al., 2021)

## Textbooks

- Applied
  Regression Analysis and Generalized Linear Models, Third Edition:
  Chapter 25 (draft) Bayesian Estimation of Regression Models (Fox,
  2022)
- Bayesian
  Data Analysis (Gelman et al., 2013)
- Bayes Rules!: An
  Introduction to Applied Bayesian Modeling (Johnson & Dogucu,
  2022)
- Doing
  Bayesian data analysis: A tutorial with R, JAGS, and Stan, second
  edition (Kruschke, 2015)
- Doing
  Bayesian Data Analysis in brms and the tidyverse, chapter 8 (Kurz,
  2023)
- Statistical
  Rethinking: A Bayesian Course with Examples in R and STAN (McElreath,
  2020)

## Software

- brms: An R
  Package for Bayesian Multilevel Models Using Stan (Bürkner,
  2017)
- Doing
  Bayesian data analysis: A tutorial with R, JAGS, and Stan, second
  edition (Kruschke, 2015)
- Doing
  Bayesian Data Analysis in brms and the tidyverse, chapter 8 (Kurz,
  2023)

## Understanding Priors and Their Impact (e.g., Sensitivity Analysis, Prior Predictive Check)

- The
  Importance of Prior Sensitivity Analysis in Bayesian Statistics:
  Demonstrations Using an Interactive Shiny App (Depaoli et al.,
  2020)
- Doing
  Bayesian data analysis: A tutorial with R, JAGS, and Stan, second
  edition (Kruschke, 2015)
- Influence
  of Priors: Popularity Data (Smeets & van de Schoot, 24 August,
  2019)

## Bayesian Model Evaluation and Selection

- Using
  leave-one-out (LOO) cross-validation and widely applicable information
  criterion (WAIC) (Vehtari et al., 2016)
- Bayesian
  Model Averaging (Hinne et al., 2020)
- Bayesian
  Model Averaging: A Tutoria (Hoeting et al., 1999)
- Objective
  Bayesian Methods for Model Selection: Introduction and Comparison
  (Berger & Pericchi, 2001)
- Using
  Stacking to Average Bayesian Predictive Distributions (Yao et al.,
  2018)

## Reporting Guidelines and Examples

- Improving
  transparency and replication in Bayesian statistics: The WAMBS-Checklist
  (Depaoli & van de Schoot, 2017)
- The
  Importance of Prior Sensitivity Analysis in Bayesian Statistics:
  Demonstrations Using an Interactive Shiny App (Depaoli et al.,
  2020)
- Bayesian
  Analysis Reporting Guidelines (Kruschke, 2021)

# Session and Packages Information

```
sessionInfo()
```

```
## R version 4.4.1 (2024-06-14)
## Platform: aarch64-apple-darwin20
## Running under: macOS 15.1
## 
## Matrix products: default
## BLAS:   /Library/Frameworks/R.framework/Versions/4.4-arm64/Resources/lib/libRblas.0.dylib 
## LAPACK: /Library/Frameworks/R.framework/Versions/4.4-arm64/Resources/lib/libRlapack.dylib;  LAPACK version 3.12.0
## 
## locale:
## [1] en_US.UTF-8/en_US.UTF-8/en_US.UTF-8/C/en_US.UTF-8/en_US.UTF-8
## 
## time zone: America/Toronto
## tzcode source: internal
## 
## attached base packages:
## [1] stats     graphics  grDevices utils     datasets  methods   base     
## 
## other attached packages:
##  [1] tidybayes_3.0.6        lubridate_1.9.3        forcats_1.0.0         
##  [4] stringr_1.5.1          dplyr_1.1.4            purrr_1.0.2           
##  [7] readr_2.1.5            tidyr_1.3.1            tibble_3.2.1          
## [10] tidyverse_2.0.0        misty_0.6.4            haven_2.5.4           
## [13] GGally_2.2.1           ggplot2_3.5.1          car_3.1-2             
## [16] carData_3.0-5          brms_2.21.0            Rcpp_1.0.13           
## [19] bayestestR_0.13.2      bayesplot_1.11.1       BayesFactor_0.9.12-4.7
## [22] Matrix_1.7-0           coda_0.19-4.1         
## 
## loaded via a namespace (and not attached):
##  [1] pbapply_1.7-2        gridExtra_2.3        inline_0.3.19       
##  [4] rlang_1.1.4          magrittr_2.0.3       ggridges_0.5.6      
##  [7] matrixStats_1.3.0    compiler_4.4.1       mgcv_1.9-1          
## [10] loo_2.7.0            png_0.1-8            callr_3.7.6         
## [13] vctrs_0.6.5          reshape2_1.4.4       pkgconfig_2.0.3     
## [16] arrayhelpers_1.1-0   fastmap_1.2.0        backports_1.5.0     
## [19] labeling_0.4.3       utf8_1.2.4           rmarkdown_2.28      
## [22] tzdb_0.4.0           ps_1.7.6             MatrixModels_0.5-3  
## [25] xfun_0.47            cachem_1.1.0         jsonlite_1.8.9      
## [28] highr_0.11           parallel_4.4.1       R6_2.5.1            
## [31] bslib_0.8.0          stringi_1.8.4        RColorBrewer_1.1-3  
## [34] StanHeaders_2.32.8   jquerylib_0.1.4      tufte_0.13          
## [37] rstan_2.32.6         knitr_1.48           splines_4.4.1       
## [40] timechange_0.3.0     tidyselect_1.2.1     rstudioapi_0.16.0   
## [43] abind_1.4-5          yaml_2.3.10          codetools_0.2-20    
## [46] curl_5.2.1           processx_3.8.4       pkgbuild_1.4.4      
## [49] lattice_0.22-6       plyr_1.8.9           withr_3.0.0         
## [52] bridgesampling_1.1-2 posterior_1.5.0      evaluate_1.0.0      
## [55] ggstats_0.6.0        RcppParallel_5.1.7   ggdist_3.3.2        
## [58] pillar_1.9.0         tensorA_0.36.2.1     checkmate_2.3.1     
## [61] stats4_4.4.1         insight_0.20.1       distributional_0.4.0
## [64] generics_0.1.3       hms_1.1.3            rstantools_2.4.0    
## [67] munsell_0.5.1        scales_1.3.0         glue_1.7.0          
## [70] tools_4.4.1          mvtnorm_1.2-5        grid_4.4.1          
## [73] QuickJSR_1.1.3       datawizard_0.11.0    colorspace_2.1-0    
## [76] nlme_3.1-164         cli_3.6.3            fansi_1.0.6         
## [79] svUnit_1.0.6         Brobdingnag_1.2-9    V8_5.0.1            
## [82] gtable_0.3.5         sass_0.4.9           digest_0.6.37       
## [85] farver_2.1.2         htmltools_0.5.8.1    lifecycle_1.0.4     
## [88] prettydoc_0.4.1
```
